# Washout on Contrast-Enhanced Ultrasound of Benign Focal Liver Lesions—A Review on Its Frequency and Possible Causes

**DOI:** 10.3390/diagnostics15080998

**Published:** 2025-04-14

**Authors:** Kathleen Möller, Christian Görg, Martin Krix, Christian Jenssen, Yi Dong, Xin-Wu Cui, Christoph F. Dietrich

**Affiliations:** 1 Medical Department I/Gastroenterology, SANA Hospital Lichtenberg, 10365 Berlin, Germany; k.moeller@live.de; 2 Interdisciplinary Center of Ultrasound Diagnostics, Gastroenterology, Endocrinology, Metabolism and Clinical Infectiology, University Hospital Giessen and Marburg, Philipp University of Marburg, Baldingerstraße, 35037 Marburg, Germany; goergc53@gmail.com; 3 Global Medical & Regulatory Affairs, Bracco Imaging, 78467 Konstanz, Germany; martin.krix@bracco.com; 4 Department of Internal Medicine, Krankenhaus Märkisch Oderland, 15344 Strausberg, Germany; c.jenssen@khmol.de; 5 Brandenburg Institute for Clinical Ultrasound (BICUS) at Brandenburg Medical University, 16816 Neuruppin, Germany; 6 Department of Ultrasound, Xinhua Hospital Affiliated to Shanghai Jiaotong University School of Medicine, Shanghai 200082, China; drdaisydong@hotmail.com; 7 Medical Ultrasound, Tongji Hospital of Tongji Medical College of Huazhong University of Science and Technology, Wuhan 430030, China; cuixinwu@live.cn; 8 Department General Internal Medicine (DAIM), Hospitals Hirslanden Bern Beau Site, Salem and Permanence, 3013 Bern, Switzerland

**Keywords:** benign liver lesions, contrast-enhanced ultrasound (CEUS), late phase (LP), washout, hypoenhancement

## Abstract

In all imaging methods, including contrast-enhanced ultrasound (CEUS), enhancement in the late phase (LP) is an important criterion for differentiating between benign and malignant focal liver lesions (FLLs). In general, malignant liver lesions are characterized by hypoenhancement and washout in the LP. A lesion with LP hyperenhancement or isoenhancement in the non-cirrhotic liver is usually benign. However, LP hypoenhancement in benign lesions is not so rare, and is even normal and the standard for some lesions, and there are exceptions for each tumor entity that can represent a diagnostic challenge. Knowing these contrast patterns and exceptions is key for correct diagnosis and patient management. The following narrative review describes the contrast behaviors and the frequency of washout and LP hypoenhancement for common as well as rare benign liver lesions and analyzes its causes.

## 1. Introduction

Contrast-enhanced ultrasound (CEUS) is an accurate method for characterizing liver lesions [1]. SonoVue^®^ (Bracco Imaging S.p.A. Milan, Italy), also known as Lumason^®^ in the USA, and Sonazoid^®^ are available in certain regions of the world as ultrasound contrast agents (UCAs) approved for characterization of focal liver lesions (FLL). SonoVue^®^ is a strict blood pool agent [1]. Sonazoid^®^ has an additional post vascular or so-called Kupffer cell phase [2]. The current paper exclusively refers to the UCA SonoVue^®^ since approved in Europe. The dual blood supply of the liver via the hepatic artery and portal vein is reflected by vascular phases (Table 1). 

The disappearance of the SonoVue® bubbles occurs after about 4–8 min post injection (p.i.) and is accelerated by continuous sonication [1].

The arterial phase (AP) of contrast enhancement allows description of the vascular architecture which are characteristic of the vascularity of FLL. CEUS describes wheel spoke-like, globular, rim-like enhancement, hyperenhancement, isoenhancement, and hypoenhancement of the lesion with homogeneous or inhomogeneous distribution. In the portal venous (PVP) and late phase (LP), it is important whether a lesion shows washout and is hypoenhanced compared to the surrounding liver tissue or whether iso- or hyperenhancement is still present. Washout is a hallmark of malignant FLLs and is further differentiated according to time and intensity. Early and marked washout before 60 s is typical for metastases and cholangiocellular carcinomas [3,4,5,6,7,8]. A late washout not before 60 s of lower intensity is seen in the majority of hepatocellular carcinomas in the cirrhotic liver [5,7,8,9,10]. Hyper- and isoenhancement in the LP is a characteristic of benign liver lesions [1]. However, benign liver lesions can also show washout in CEUS. This is a diagnostic challenge. The following review describes these benign lesions, the frequency and characteristics of washout, and attempts to explain it. Features of rare FLLs are described in further detail, as these lesions are often not well described. 

## 2. Method/Search Strategy

The literature search and analysis were performed specifically for benign liver lesions with hypoenhancement and washout in the LP in CEUS with SonoVue. PubMed was the database searched for entries until 31 December 2024. The keywords and binary operators used are listed in brackets. The summaries were reviewed and suitable articles were selected for further analysis. Similar articles on PubMed database were also checked for relevant abstracts.

Hemangioma AND (CEUS OR late phase on CEUS OR late phase hypoenhancement on CEUS OR washout OR SonoVue). Focal nodular hyperplasia AND (CEUS OR late phase hypoenhancement on CEUS OR late phase OR washout on CEUS OR SonoVue). Hepatocellular adenoma AND (CEUS OR late phase on CEUS OR late phase hypoenhancement on CEUS OR late phase). Hepatic tuberculosis AND CEUS; tuberculosis AND CEUS; tuberculosis AND hepatic lesions. Hepatic sarcoidosis AND CEUS; sarcoidosis AND CEUS; sarcoidosis AND hepatic lesions. Hepatic angiomyolipoma AND CEUS; hepatic epithelioid angiomyolipoma AND CEUS, hepatic PEComa AND CEUS, hepatic inflammatory lesions AND CEUS, hepatic inflammatory pseudotumor AND CEUS; liver abscesses AND CEUS, hepatic abscesses AND CEUS; amebic abscesses AND CEUS; mycotic abscesses AND CEUS; hepatic peliosis AND CEUS; cholangiocellular adenoma AND CEUS; extramedullary hematopoiesis AND CEUS.

**Figure 1:**
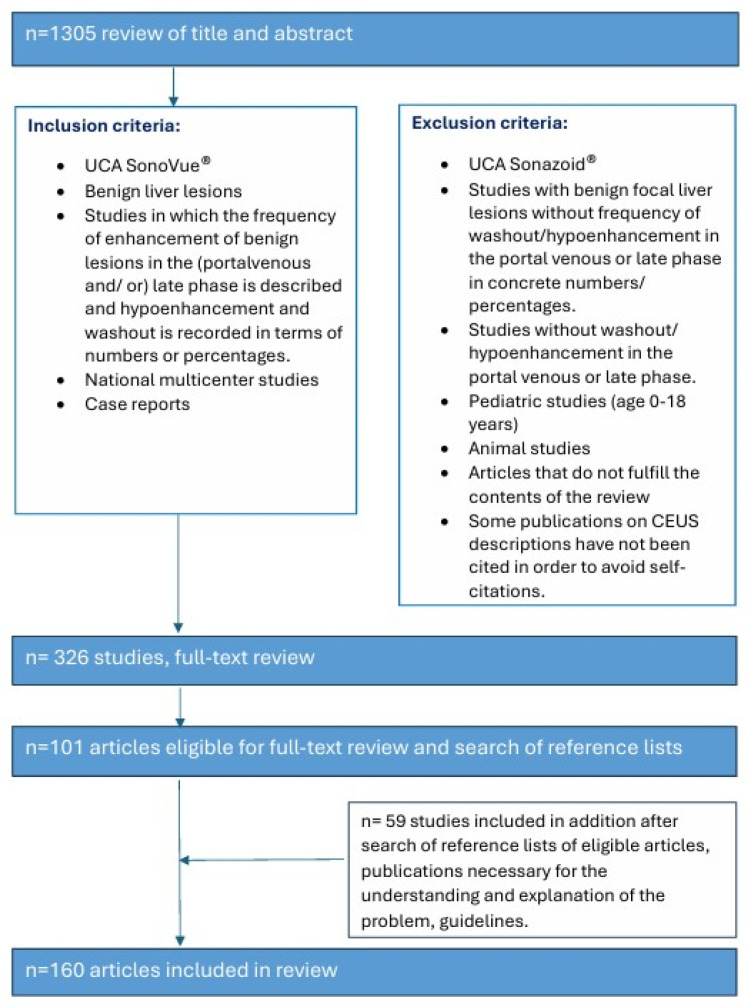
Search strategy.

Original publications with the focus on cohort studies, multicenter studies, and meta-analyses that actually refer to the topic of benign liver lesions and CEUS were identified and analyzed. Only studies in which SonoVue^®^ was used as an UCA were included. For further analysis, those studies were used to explicitly report data on the enhancement of benign lesions in the late CEUS phase. Data that documented (ideally in tabular form) the frequencies in numbers and percentages of benign FLLs with hypoenhancement in the LP were used. The timing of the washout in the vascular CEUS phases was analyzed. The results are presented for each FLL. Each section of FLL starts with a brief summary of the lesion characteristics—with the aim of understanding why a benign lesion may show hypoenhancement in the LP (Figure 1).

We consider a possible limitation to be that a histological diagnosis may not be available in all cases and CT or MRI served as a correlation. The examinations were performed in accordance with the guidelines, but on different ultrasound devices with different MI settings and contrast medium quantities.

## 3. Late Phase Washout in CEUS of Benign FLLs

A washout with hypoenhancement in the LP compared to the surrounding normal liver tissue is a typical characteristic of malignant FLLs [1,3,4]. This is based on the key concept that malignant FLLs contain a reduced amount or lack of liver tissue and a lower blood supply or no supply by the portal vein. This causes FLL hypoenhancement during LP compared to the liver in which the UCA microbubbles create a high enhancement during the sinusoidal phase. In principle, this also applies to hepatocellular carcinoma (HCC), as HCCs have a stronger arterial and a reduced portal vein supply than normal liver, depending in particular on the degree of differentiation [11,12,13,14,15]. This not only causes arterial hyperenhancement, but also hypoenhancement during LP, although milder and delayed compared to the washout observed in metastatic FLLs [1,3,4]. However, hypoenhancement in FLLs can also occur due to other reasons. In fact, washout with hypoenhancement in the LP of CEUS enhancement has also been reported for all types of benign FLLs; in some of them it is rare, while in others LP hypoenhancement is a common appearance. This washout can begin at different times, in the PVP or LP, and can vary in intensity. Rarely, the washout can also start in the late AP with an overlap to the PVP. The DEGUM multicenter study, there were 86/1349 FLLs that could not initially be assigned with CEUS, and out of them, 47 of 56 FLLs with hypoenhancement in the LP ultimately corresponded to benign lesions [16]. There were examples of benign lesions with LP hypoenhancement for a variety of benign diagnoses: focal nodular hyperplasia (FNH), hemangioma, hamartoma, lipoma, angiomyolipoma, hepatocellular adenoma (HCA), inflammatory lesion, abscess, echinococcus, regenerative nodule, hematoma, necrosis, granuloma, and other forms of scarring [16,17,18,19,20]. Lesions with hypoenhancement in the LP must be distinguished from those with non-enhancement in all phases, such as hepatic hematoma or completely sclerosed hemangioma [21]. In an analysis of 27 benign FLLs in the steatotic liver with respective hypoenhancement in the LP, inflammatory pseudotumor (IPT), focal fatty change, parasite-caused infection, and hemangioblastoma were included in addition to the entities already mentioned [22]. Table 2 summarizes the key findings reported in the literature related to washout or hypoenhancement in the LP of the three most relevant FLLs, i.e., hemangioma, FNH, and hepatocellular adenoma (HCA). The details for each benign FLL entity are described in the following section. 

## 4. Various Common and Rare Benign FLLs with Washout and LP Hypoenhancement

### 4.1. Hemangioma

Hemangiomas are hamartomas in the most general sense. The most common type of liver hemangioma consists of cavernous vascular cavities. These are lined with a single layer of endothelium, separated by fibrous septa and filled with blood. Due to the very slow flow, thrombi can form in the vascular cavities and lead to partial or complete thrombosis. Collagenous scars can form as a result of thrombosis [23]. 

Typical enhancement patterns of hemangiomas in CEUS are peripheral nodular (synonymously: peripheral globular) enhancement (I), peripheral circular enhancement (II) both with centripetal filling, or rapid diffuse enhancement in the AP (III) (so-called high-flow hemangiomas) [23,24,25,26,27]. Centripetal enhancement can be slower or faster, complete or incomplete. With slow flow, complete filling can continue into the LP. In the case of so-called shunt or high-flow hemangiomas, filling occurs so quickly that no pattern can be detected by the eye. This can make differential diagnosis difficult. Hemangiomas have also been divided into six different vessel types according to their architecture [1,24,28,29]: type I corresponds to the classic type; type II contrasts very fast and has a rapid inflow; type III shows arteriosystemic fistulas (shunt hemangioma); type IVA and IVB have proximal or distal portovenous anastomoses; type V has both arteriosystemic and portovenous anastomoses. These fistulas are associated with perilesional enhancement [28]. This can be recognized by an extensive enhancement of the surrounding liver parenchyma in the AP. Arterioportal shunts are detected in up to 26% of hemangiomas. These are mostly small hemangiomas and usually with high flow [30]. Such high flow hemangiomas are, in turn, often hypoechoic in B-mode US [31].

Atypical hemangiomas include very large “giant” hemangiomas, which are usually associated with cavernous thrombosis and do not completely enhance. Sclerosed hemangiomas develop as a result of thrombosis of cavernous hemangiomas [32]. In this case, enhancement is absent in all vascular phases. However, this is not a washout, but an atypical hemangioma due to complete sclerosis. 

The washout of hemangiomas is mostly seen very late, only rarely in the PVP [4,33]. In a study by Bhayana et al., washout was predominantly (5/6) seen after 180 s [4]. Gianetti et al. reported seven atypical hemangiomas with washout [33]. This occurred predominantly after 160 s. The common feature of the hemangiomas with washout was a peripheral location, in some cases near Glisson’s capsule [33]. Fang et al. described hemangiomas of “Fast-in-fast-out-type“ [27]. These were <30 mm, with mild fade in the PVP and hypoechoic in the LP. Various authors attribute the decrease in contrast enhancement with the development of LP hypoenhancement to intratumoral arteriosinusoidal shunts [4,27,33]. This was based on the detection of perinodular vascularity as well as perinodular enhancement [33]. 

**Figure 2:**
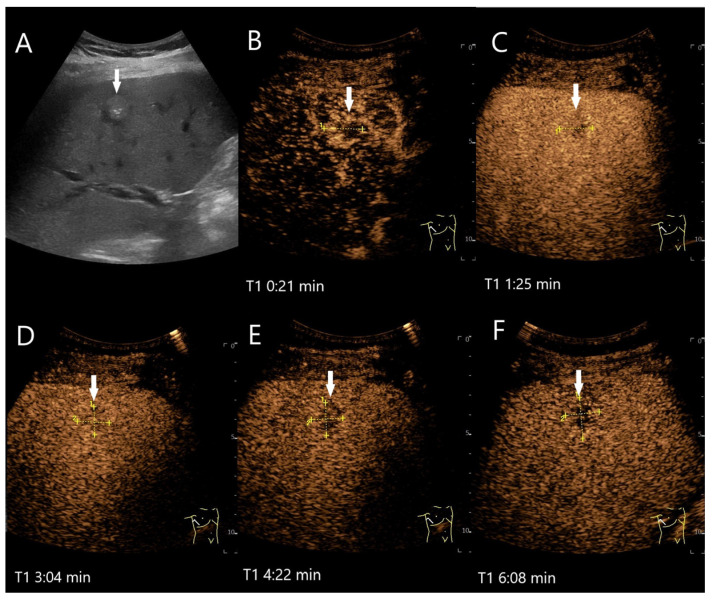
Partially fibrosed hemangioma. As part of a staging examination for adenocarcinoma of the gastro-esophageal junction, a 19 mm, smoothly bordered hyperechoic lesion (arrow) with an implied hypoechoic rim and punctate echogenic reflexes is diagnosed in the liver (**A**). On CEUS, the small lesion (between the markings) shows a marginal contrast image that is not completely smooth (**B**). In the PVP after 1:25 min, most of the lesion is enhanced, but slightly less than the surrounding parenchyma and with a small portion that is clearly hypoenhanced (**C**). After 3:04 min, the lesion is slightly hypoenhanced (**D**), while after 4:22 min (**E**) and 6:08 min (**F**), the lesion shows an unequivocal hypoenhancement. The lesion did not clearly correspond to a hemangioma on computed tomography either. This was the reason for a US-guided biopsy. Histology revealed a hemangioma, partially fibrosed and with tiny calcifications.

**Figure 3:**
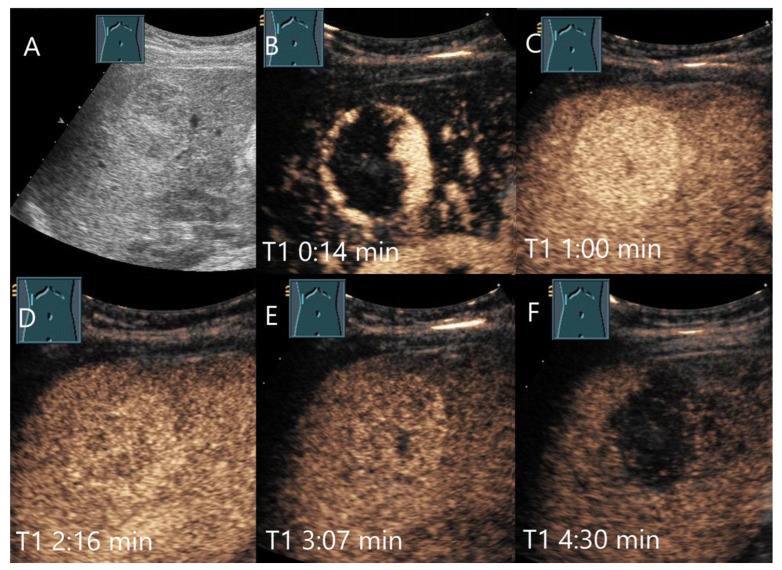
Cavernous hemangioma. Female patient. Incidental finding of a 40 × 32 mm heterogeneous hypoechoic FLL (**A**). The CEUS shows arterial marginal hyperenhancement after 14 s (**B**) and homogeneous AP hyperenhancement after 1:00 min (**C**). Decreasing heterogeneous hyperenhancement after 2:16 min (**D**) and 3:07 min (**E**). After 4:30 min (**F**), there is a clear hypoenhancement. Clinical ultrasound revealed the diagnosis of a hemangioma. Histologically, a cavernous hemangioma was confirmed.

Various causes are discussed for washout in hemangiomas with hypoenhancement in the LP. The first one is caused by a technical aspect: UCA bubbles can be destroyed by too intense and prolonged exposure to ultrasound waves. If the ultrasound power (characterized by the Mechanical Index, MI) has been set too high, this further contributes to UCA bubble destruction. If hemangiomas fill up more slowly than in the surrounding liver parenchyma, the degree of disruption may be greater than the replenishment. It is, therefore, recommended that the ultrasound examination is not carried out continuously up to the 5th minute, but intermittently [16]. If an FLL possibly represents a hemangioma, we prefer a video loop in the AP and then every 30 or 60 s in the PVP and LP. However, in our experience, there are hemangiomas that still show a washout even with this procedure. A mild fade in the PVP and hypoenhancement in LP are described for hemangiomas with rapid onset [27]. In hemangiomas with rapid filling, it is debated whether the rapid filling has an equally rapid outflow, which in turn is more pronounced than the contrasting of the surrounding liver parenchyma [4]. Finally, fibrosis or scars lead to hypoenhancement during all contrast phases. Fibrosis in any type of lesion reduces the sinusoidal network, and thus, the vascular volume [4]. Hemangioma with a faint washout, in which fibrotic lesions have been described histologically, is demonstrated in Figure 2. A pronounced hypoenhancement in the LP of a hemangioma is shown in Figure 3. 

### 4.2. Focal Nodular Hyperplasia (FNH)

FNH is not a true tumor, but a hamartoma or regenerative nodule resulting from a “vascular anomaly”. The FNH contains normal liver structures but with a different architecture. A typical feature is a strong feeding artery which is located centrally or, more rarely, peripherally in the FNH and is surrounded by scar tissue. Arterial blood flow in FNH drains from the abnormal arteries via capillaries into sinusoids adjacent to fibrous septa. Blood in the sinusoids then drains into the hepatic vein either directly or via perinodular sinusoids. The portal vessels, therefore, appear to be completely excluded from the blood circulation of FNH [34,35,36,37,38]. 

Typical characteristics in CEUS are feeding artery, spoke wheel sign, centrifugal arterial enhancement with transient peripheral unenhanced zone, homogeneous hyperenhancement in the AP, hyper- or isoechoic enhancement, and central hypoechoic scar during the LP [25,39,40,41,42,43,44,45]. Further subtypes are FNH with central artery with wheel-spoke-like centrifugal enhancement, FNH with an eccentric, laterally branching feeding artery, and the telangiectatic variant. While Bhayana et al. [4] described washout in up to 29% of all FNH, this was observed much less frequently in all other studies (Table 2) or not at all [44]. The onset of washout in FNH is described later than 75 s p.i. in 78% of nodules. Only 22% showed a washout at an earlier time; the time interval was given as 30–75 s [4].

Washout is not linked to the size of the FNH [43,46,47]. Hypoenhancement in the PVP was described in 10% of FNHs ≤ 35 mm and 8% of FNH > 35 mm [43]. In this study, all FNHs with LP hypoenhancement were observed in a steatotic livers [43]. In a further comparative study of FNH in steatotic and non-steatotic livers, 21% of FNH with steatotic liver background showed hypoenhancement in the LP, and 0% with non-steatotic liver [48].

In a quantitative analysis of CEUS in benign FLLs with LP hypoenhancement it was found that the enhancement duration of liver parenchymal background was significantly longer in fatty liver (300 ± 17 s) than in non-steatotic liver (213 ± 11 s, *p* < 0.05). From this, it can be inferred that the UCA persists more intensively and longer in fatty liver tissue than in normal liver tissue. This could mean that the FNHs are hypoenhanced only in relation to the longer enhanced steatotic liver [22].

In further research, wheel-spoke-like arterial contrast enhancement correlated negatively with the size of the FNH, but there was no correlation with washout in the LP. The FNHs with washout were located in both steatotic and non-steatotic liver. In steatotic liver, the authors considered a pseudo washout; in the non-steatotic liver, the washout was true [47].

Like in other tumors, and already described for hemangiomas, regressive changes with the development of tumor fibrosis can occur in FNH in the course of the disease and be the cause of hypoenhancement. An analysis of the size progression of FNH in 450 female patients indicated that the size behavior shows a convex course, starting with initial growth until menopause was reached. After menopause, in most cases, a decrease in size with involution occurred [49]. On color Doppler imaging (CDI), after 7.0 ± 2.6 years, almost no vascular signals were detectable in the lesions. This was interpreted as a natural obliteration tendency of the arteries in FNH, with the consequence of shrinkage of the FNH [50]. However, if obliteration of the arterial vessels with regressive changes in the FNH are the cause of a washout, then it would be expected that the lesions would also be hypoenhanced in the AP. However, this is not usually described. It can be deduced from this that size as an absolute parameter [43] is not a measure of possible hypoenhancement in the LP, but rather the individual decrease in size over time.

As a rule, patients with confirmed FNH and without symptoms do not undergo close monitoring of the lesion. If a patient has had FNH for a long time and the lesion shows a washout in the course of the disease, this can be due to regressive changes. Simultaneous size regression is a sign of benignity. The most common question raised by imaging is the differential diagnosis between FNH and HCA [4,39,41,42,43,51]. 

HCA and FNH have typical characteristics in the AP. Nevertheless, the typical characteristics are not developed in all FNH and all HCA [25,42,43,51]. However, HCA tend to wash out in the late PVP and LP. As different treatment or surveillance strategies arise in both lesions, washout is always a diagnostic challenge, usually resulting in histologic confirmation. In rare cases, metastases can also show a wheel-spoke-like enhancement in the AP [25]. This is then associated with a rapid metastasis-typical washout in the PVP. A biopsy must also be considered in this case.

**Figure 4:**
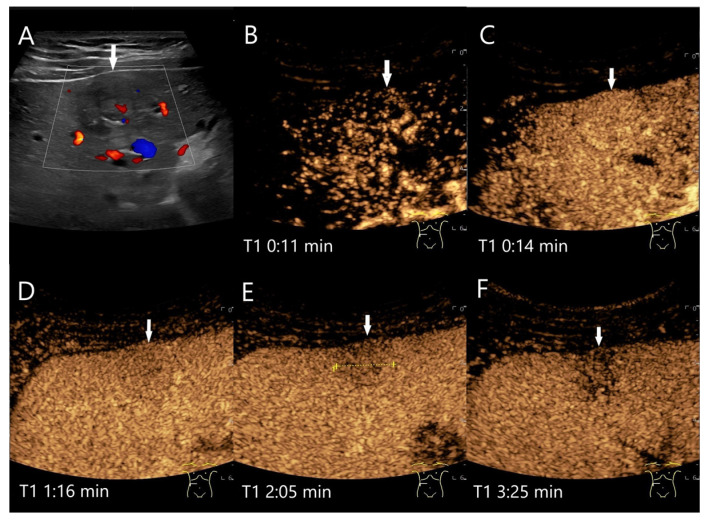
FNH. Incidental finding as part of the diagnosis of choledocholithiasis. 20 mm large, slightly hypoechoic lesion (arrow) with contour protrusion of the liver, centrally visible vessel on color Doppler imaging (CDI) (**A**). In the CEUS, wheel-spoke-like central enhancement (**B**) and homogeneous hyperenhancement in the AP (**C**) is seen. In the PVP after 60 s, a very shallow washout begins (**D**). This continues in the LP at 2:05 min (**E**) and is very pronounced after 3:25 min (**F**). This was the reason for a US-guided biopsy. The histology was consistent with FNH with focal fibrosis.

**Figure 5:**
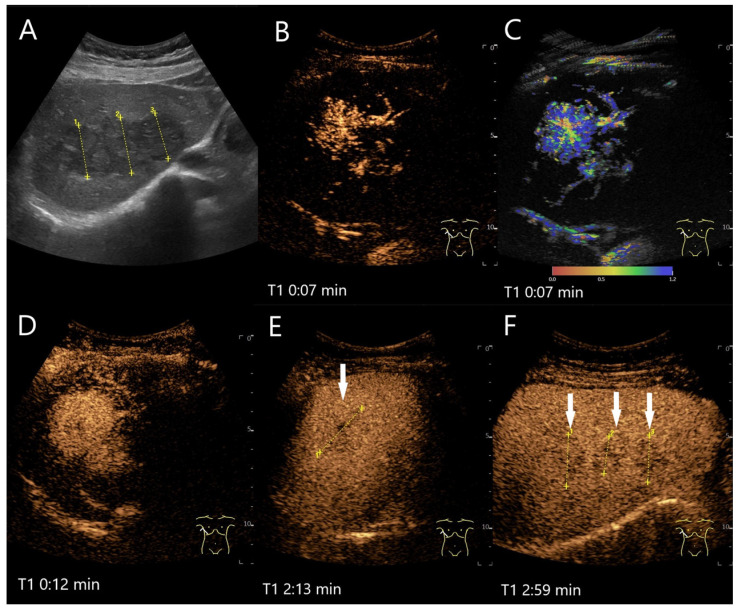
FNH/nodular regenerative hyperplasia newly diagnosed in follow-up care after colon carcinoma and adjuvant chemotherapy with capecitabine and irinotecan. Initially, the lesions with a size up to 30 mm showed a wheel-spoke-like homogeneous hyperenhancement in the AP with isoenhancement in the PVP and LP. Over time, however, hypoenhancement developed in the LP. The images show the lesions three years after the initial diagnosis: the lesions (between the markings) are slightly larger (**A**). CEUS (**B**) and parametric imaging (**C**) show a typical wheel-spoke-like enhancement. In parametric imaging (**C**), the time of enhancement is displayed in different colors. The lesion is homogeneously hyperenhanced in the AP, a central scar is visible (**D**). In the LP, the lesions (arrows) are hypoenhanced with emphasis on the central parts (**E**,**F**). These lesions were, therefore, not clearly classifiable as benign, particularly in a patient with a history of colon carcinoma. A US-guided biopsy is performed. This revealed findings compatible with FNH and no evidence of metastases.

Figure 4 and Figure 5 demonstrate that hypoenhancement can cause diagnostic difficulties, particularly in patients with a history of tumors. Table 2 summarizes various studies with the frequencies of hypoenhancement in hemangiomas, FNH and HCA. As a rule, hypoenhancement is less frequently observed in FNH than in HCA.

**Table 2:** Frequency of LP hypoenhancement in hemangiomas, focal nodular hyperplasia, and hepatocellular adenoma.

Study	FLL (***n***)	LP Washout	Comments
Ding 2005 [52]	Benign lesions *n =* 51	*n =* 11/51 (22%)	
	Hemangioma *n =* 27	*n =* 3/27 (11%)	
	FNH *n =* 16	*n =* 2/16 (12.5%)	
Kim 2008 [41]	FNH *n =* 43	PVP: Reader 1: 6/43 (14%) Reader 2: 4/43 (9%)	Two readers, hypoenhancement in PVP, no data about LP.
	HCA *n =* 19	PVP: Reader 1: 10/19 (53%) Reader 2: 7/19 (37%)	Two readers, hypoenhancement in PVP, no data about LP.
Strobel 2009 [25] DEGUM-Multicenter-Study	Hemangioma *n =* 242	22.9%	The frequency of hypoenhancement is given as a percentage in this paper. The total number of FNH and hemangiomas allows conclusions to be drawn about the number of patients.
	FNH *n =* 170	6.4%	
Piscaglia 2010 [46]	FNH *n =* 90	*n =* 3/90 (3.3%)	“Faintly” hypoechoic.
Bhayana 2010 [4]	Hypervascular benign FLL *n =* 74 (overall *n =* 146 FLL)	36% of all benign FLL	Washout occurred in 36% of benign and 97% of malignant FLL. The onset of washout after injection was defined as <30 s/after 30–75 s/75–180 s/<180 s.
	Hemangioma	*n =* 6/29 (21%)	Mostly (83%) > 180 s.
	HCA	*n =* 5/7 (71%)	Mostly (71%) 75–180 s.
	FNH	*n =* 9/31 (29%)	Mostly (56%) 75–180 s.
Wang 2013 [53]	FNH *n =* 85	*n =* 22/85 (26%)	In 18%, the hypoenhancement was present in PVP.
Bertin 2014 [47]	FNH *n =* 94	*n =* 5/94 (5.3%)	Start of washout in the PVP *n =* 1/5 (20%). Start in the LP *n =* 4/5 (80%).
Roche 2015 [43]	FNH *n =* 43	Reader 1: 4/43 (9%) Reader 2: 4/43 (9%)	31% of all FNH with concomitant steatosis showed washout from the PVP onwards. All FNH with washout had steatosis hepatis at the same time. There was no relation to the size of the lesion. The washout is described as portal venous. Two readers.
	HCA *n =* 20	Reader 1: 9/20 (45%) Reader 2: 7/20 (35%)	Hypoenhancement from the PVP was more frequent with HCA > 35 mm (83%) than < 35 mm (29%). The washout is described as portal venous. Two readers.
Kong 2015 [42]	FNH *n =* 28	*n =* 3/28 (11%)	
	HCA *n =* 10	*n =* 6/10 (60%)	
Taimr 2017 [51]	FNH *n =* 181	*n =* 8/181 (4%)	
	HCA *n =* 143	*n =* 21/143 (15%)	
Fang 2019 [27]	Hypoechoic hepatic hemangioma *n =* 101	Hypoenhanced or mild fade *n =* 6/101 (6%)	Center mild fade in PVP in 4/101 (4%).

### 4.3. Hepatocellular Adenoma

Hepatocellular adenoma (HCA) is a mostly benign tumor, although the vast majority of HCAs show a washout (Table 3), and thus, cause differential diagnostic problems. Large HCAs (≥50 mm) are associated with bleeding (15–20%) and a low probability of malignant transformation into hepatocellular carcinoma (HCC; 1.6%) [54,55]. Those two complications are rare in HCAs < 50 mm [54,55]. In the International guidelines [56,57], surgical resection in female patients is only recommended for HCAs larger than 50 mm; otherwise, lifestyle corrections (cessation of contraceptives and weight reduction in the case of obesity) are initially recommended for 6 months. Male patients, in turn, have an increased risk of malignant transformation per se and a higher prevalence of the beta-catenin-activated HCA subtype (ß-HCA) [55,56,57].

Histopathologically, HCA is characterized by scattered thin-walled ducts within the tumor and the absence of portal and central veins and bile ducts or connective tissue. Depending on the subtype of HCA, varying degrees of hepatic steatosis, inflammatory cells, bile duct proliferation, hemorrhage, or dystrophic blood vessels have been described [58]. 

Current HCA classification includes six molecular subtypes of HCA: HNF1A-inactivated HCA (H-HCA), inflammatory HCA (I-HCA), β-catenin exon 3 mutated HCA (bex3HCA), β-catenin exon 7/8 mutated HCA (bex7,8HCA), Sonic Hedgehog activated HCA (shHCA), and a further approximately 10% unclassified HCA (U-HCA). β-catenin exon 3 mutated HCAs are at risk of malignant transformation [59,60]. The different HCA subtypes appear to be morphologically distinct. This is important for their appearance on imaging. In a previous histological classification without the current molecular subclassification, I-HCA was characterized by sinusoidal dilatation, abortive portal tracts, a more or less distinct inflammatory ductular reaction, and bare arteries. Steatotic HCA (now HNF1A-inactivated HCA) was characterized without sinusoidal dilatation/telangiectasia and inflammation, but by mostly diffuse steatosis/fatty degeneration of the adenoma cells [61]. Dietrich et al. described that in both the mutation-associated subtypes and the other “unclassified” HCAs, inflammatory changes and telangiectatic features can be expressed [58]. In our opinion, the differentiation between telangiectatic FNH and I-HCA is very indistinct. However, if these FLLs show hyperenhancement in the LP, CEUS is able to address the most relevant question by proving their benign nature. In CEUS, the different subtypes show different characteristics. CEUS is one of the imaging techniques used to differentiate HCA from FNH and HCC or other well-vascularized tumors. The combination of different ultrasound features also allows conclusions to be drawn about the possible subtype.

**Figure 6:**
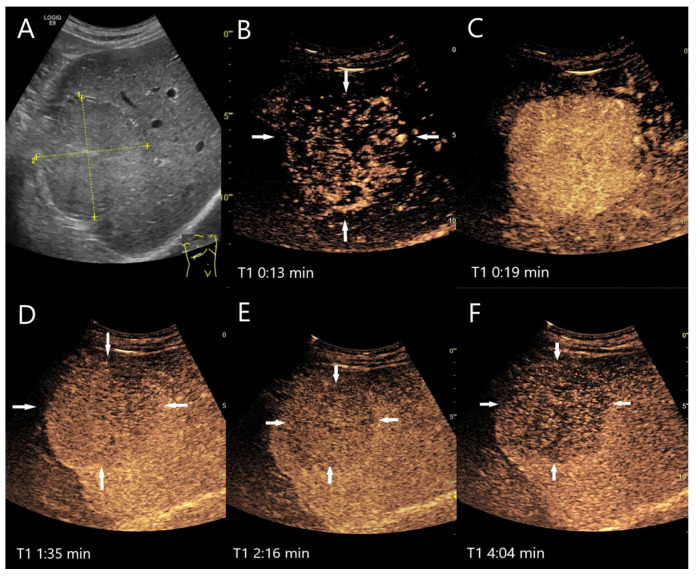
Inflammatory hepatocellular adenoma. Large mass (between the arrows) in the right lobe of the liver in a male patient (**A**). In the AP, the lesion shows a diffuse reticular enhancement (**B**), then a homogeneous hyperenhancement (**C**). In the PVP at 1:35 min, a shallow washout begins (**D**), which continues progressively in the LP (**E**,**F**). Histology after surgical resection confirmed the HCA.

**Figure 7:**
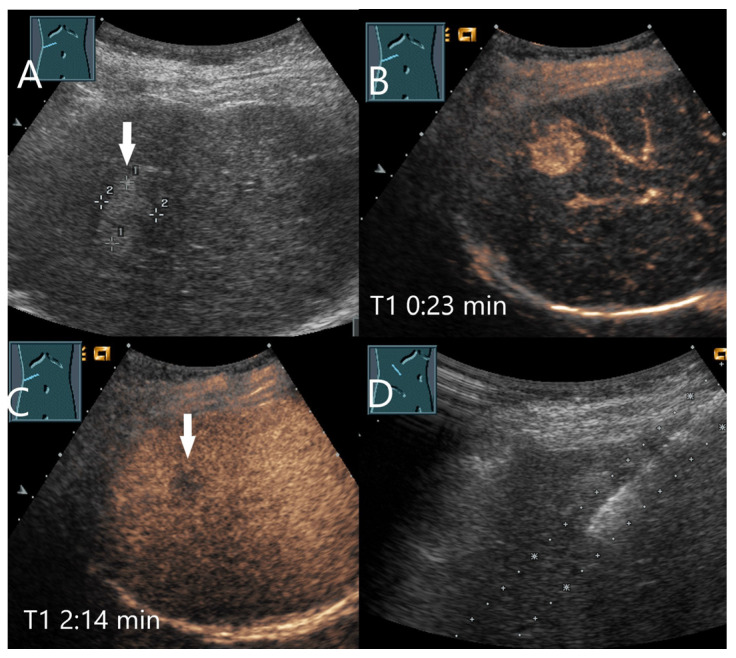
ß-catenin-mutated HCA. Female patient. Incidental findings of a 19 × 17 mm hyperechoic FLL on B-mode US (arrow) (**A**). CEUS shows arterial hyperenhancement after 23 s (**B**). The center is less hyperenhanced. After 2:14 min, the lesion shows a slight washout (arrow) (**C**). The US-guided biopsy (**D**) revealed the diagnosis of a ß-catenin mutated HCA.

Typical appearances on CEUS are centripetal or mixed/diffuse filling, and iso- or hypoenhancement during the LP [25,42,43,51]. If hypoenhancement is present in the LP, it can begin either in the PVP or in the LP. Only a few of the I-HCAs (12%) have sustained hyperenhancement at the LP. Mostly, the washout started in the time interval from 75 up to 180 s (83%) and only rarely from 30 s up to 75 s (17%) [4]. In contrast to FNH, washout in the PVP is size-dependent and was more frequent in HCAs > 35 mm (83%) than in HCAs ≤ 35 mm (29%) [43]. The time of onset of hypoenhancement did not differ significantly between HCA and FNH (with late hypoenhancement) [42]. In a study with a total of 38 HCA patients, washout in LP occurred significantly more frequently in I-HCA (65%), while less frequently in U-HCA (25%) and H-HCA (12%) [62]. In a study with a total of 53 HCAs, the majority of the numerically strongest fraction of I-HCAs (61.3%) also showed a washout. However, the much smaller group of H-HCA (7/8; 85%) was also hypoenhanced in the LP [63] (Table 3). The I-HCAs appear to be a histologically heterogeneous group. It is reported that the imaging features of I-HCAs are closely correlated with the severity of inflammatory infiltrates and telangiectasias. Typical I-HCAs with histomorphological proven pronounced inflammatory infiltrates and telangiectasias showed a persistent enhancement in the LP, while atypical I-HCAs with less pronounced telangiectasias and inflammatory infiltrates showed a washout in the PVP and in the LP [61]. A characteristic feature of I-HCA in the LP was a peripheral rim with sustained enhancement and central hypoenhancement. The central washout with sustained peripheral enhancement was not considered a malignancy criterion [62]. In an Italian multicenter study, 58% of all HCAs was hypoenhanced at the LP [64] and the sign of sustained enhancement was present only in 1/19th (5.3%). However, this feature has not been identified in further studies. Even if an arterial rim enhancement of the metastases usually results in a washout of the rim, we believe that caution is still required. The characteristics in B-mode, CDI, and CEUS allow conclusions to be drawn about the subtype for some HCAs [62]. However, in cases of doubt, histological confirmation must be carried out for arterially well-vascularized FLL without a recognizable specific vascular architecture and a washout and the PVP and/or LP. Examples of HCA with hypoenhancement in the LP are presented in Figure 6 and Figure 7.

**Table 3:** HCA subtypes and LP hypoenhancement.

Study	FLL (***n***)	LP Washout	Comments
Laumonier 2012 [62]	All HCA *n =* 38	*n =* 14/38 (37%) of all HCA	PVP was defined as the interval between 45 and 70 s, and the late PVP was observed up to 5 min after injection.
	H-HCA *n =* 16	*n =* 2/16 (12%)	In 56%, hypoenhancement started in PVP.
	I-HCA *n =* 17	*n =* 11/17 (65%)	In 12%, hypoenhancement started in PVP.
	U-HCA *n =* 4	*n =* 1/4 (25%)	In this one case, the hypoenhancement started only in the LP, not PVP.
	ß-Catenin–Activated HCA *n =* 1	*n =* 0/1 (0%)	This kind of HCA has an increased risk of malignancy but was without hypoenhancement.
Garcovic 2019 [64] Italian multicenter study	All HCA *n =* 19	*n =* 11/19 (58%)	
	I-HCA *n =* 14	*n =* 7/14 (50%)	In 3 HCAs, washout started in PVP, in 4 in the LP.
	β-catenin-activated HCA *n =* 1	*n =* 1/1 (100%)	The washout started in the PVP.
	U-HCA *n =* 4	*n =* 3/4 (75%)	In 2 HCAs, the washout started in the PVP, in 1 in the LP.
Chen 2020 [63]	All HCA *n =* 53	*n =* 28/53 (52.8%)	Start of hypoenhancement in PVP: *n =* 22/53 (41.5%); *n =* 11/53 (20.8%) central hypoenhanced area.
	H-HCA *n =* 12	*n =* 1/12 (8.3%)	Start of hypoenhancement in PVP; no central hypoenhanced area.
	ß-catenin activated HCAs *n =* 8	*n =* 7/8 (87.5%)	Start of hypoenhancement in PVP: *n =* 6/8 (75%); *n =* 1/8: central hypoenhanced area.
	I-HCAs *n =* 31	*n =* 19/31 (61,3%)	Start of hypoenhancement in PVP: *n =* 14/31 (45.2%); *n =* 9/31 (29%) central hypoenhanced area.
	U-HCAs *n =* 2	*n =* 1/2 (50%)	Start of hypoenhancement in PVP: *n =* 1/2 (50%); *n =* 1/2 central hypoenhanced area).

### 4.4. Inflammatory Lesions

Inflammatory FLLs include pyogenic and mycotic liver abscesses, parasitic lesions, granulomatous inflammation and inflammatory pseudotumors (IPT). Inflammatory lesions are an important mimicker of metastases, as they usually show a rapid and prominent washout [4] (Table 4). In a study of 56 inflammatory FLL, 80% showed hyperenhancement in the AP and 80% showed hypoenhancement in the PVP and LP [65]. Hypoenhancement in LP occurred for 81% of pyogenic abscesses and 100% of infected granulomas and IPT [65]. A comparative study between inflammatory FLLs and malignant lesions examined the contrast behavior on CEUS. The inflammatory lesions included pyogenic, parasitic, and chronic liver abscesses, IPT, and granulomatous inflammation. In PVP, 68% of the inflammatory lesions showed hypoenhancement and in LP 84%. As the proportion of HCC in the group of malignant lesions was very high, washout began earlier in the inflammatory lesions than in the malignant lesions [66]. Consideration of clinical data and obtaining histologic and microbiologically evaluable material are, therefore, important for the diagnosis.

#### 4.4.1. Bacterial (Pyogenic) Liver Abscesses

**Figure 8:**
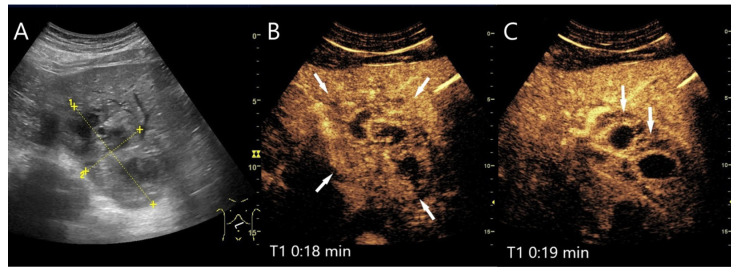
Pyogenic liver abscess. Male patient with right-sided upper abdominal pain and fever, but no previously known focus of infection. 95 × 52 mm inhomogeneous lesion in the liver (between the markings) with peripheral hypoechoic and central anechoic lesions (**A**). On CEUS, the lesion is hyperenhanced and central non-enhanced areas are demarcated (**B**). While the non-enhanced areas have a hyperenhanced rim, the initial hyperenhanced parts already show a washout in the AP and are hypoenhanced at a very early stage (arrows) (**C**). The findings corresponded to an abscess of unknown origin. Pus was obtained for microbiology during US-guided aspiration—proof of pathogenic *E. coli*.

The most common pathogens causing pyogenic abscesses are Escherichia coli and Klebsiella pneumoniae [69]. The consequences of bacterial inflammation are focal arterial hyperemia and the formation of thromboses of the small hepatic and portal veins, as well as pylephlebitis of the small portal venous vessels. Hyperemia is caused by the release of cytokines and an increased arterial perfusion due to thrombosis of the small portal venous and liver veins. In the AP, inflamed hyperemic non-necrotic areas are hyperenhanced. In the PVP and LP, the portal venous and venous thromboses lead to washout [65,69,70,71,72,73,74] (Figure 7 and Figure 8). A classification by Kunze et al. [69] describes the appearance of liver abscesses in B-mode and CEUS in their various stages of development I–IV (with subdivisions) [69]. The washout in abscesses is seen in the initially hyperemic phlegmonous segment, in the liver tissue around the liquidized necroses, in the septa, and to a lesser extent in an already formed fibrous capsule. A simplified classification was proposed by Francica et al. [68,75]. 

**Figure 9:**
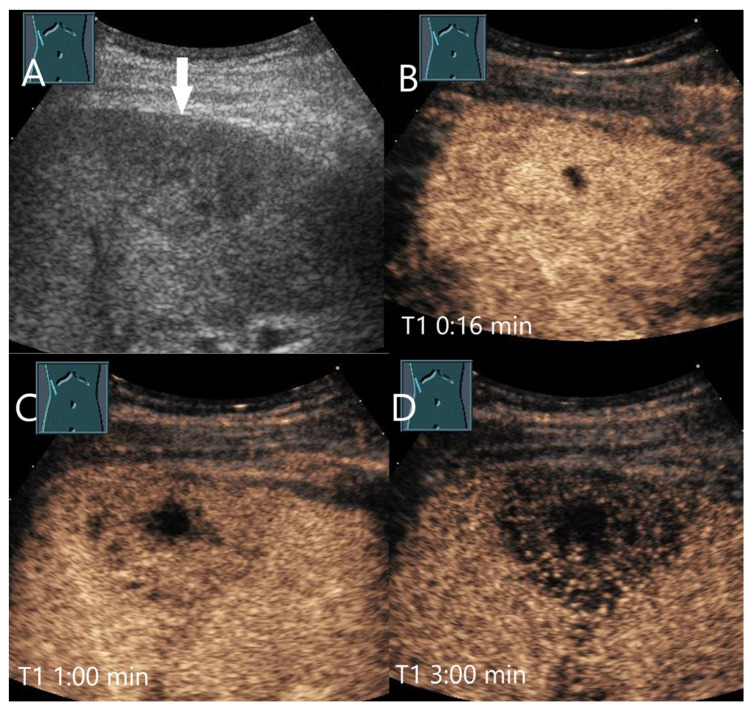
Chronic pyogenic liver abscess. Patient with hypoechoic lesion (arrow) with high inflammatory laboratory (**A**). CEUS shows mild arterial hyperenhancement after 16 s (**B**), with mild washout after 1 min (**C**) and progressive washout after 3 min (**D**). The US-guided biopsy revealed the histologic diagnosis of a chronic granulocytic sclerosing inflammation.

The washout of a large, increasingly hyperenhanced rim around a central necrosis or capsule should not be confused with necrotically disintegrating liver metastases, cystic solid metastases, or necrotically decaying tumors. Nevertheless, these differential diagnoses should not be ignored [76]. As a rule, the clinical and paraclinical signs of infection are indicative. Phlegmonous inflammation can be hypoechoic in the B-mode, arterially hyperenhanced in the CEUS, and show a rapid washout in the PVP and LP (Figure 8 and Figure 9). Differentiation from liver metastases can be very difficult if based only on CEUS features. The arterial hyperenhancement of the perilesional liver parenchyma may be indicative of inflammation.

#### 4.4.2. Mycotic Abscesses

**Figure 10:**
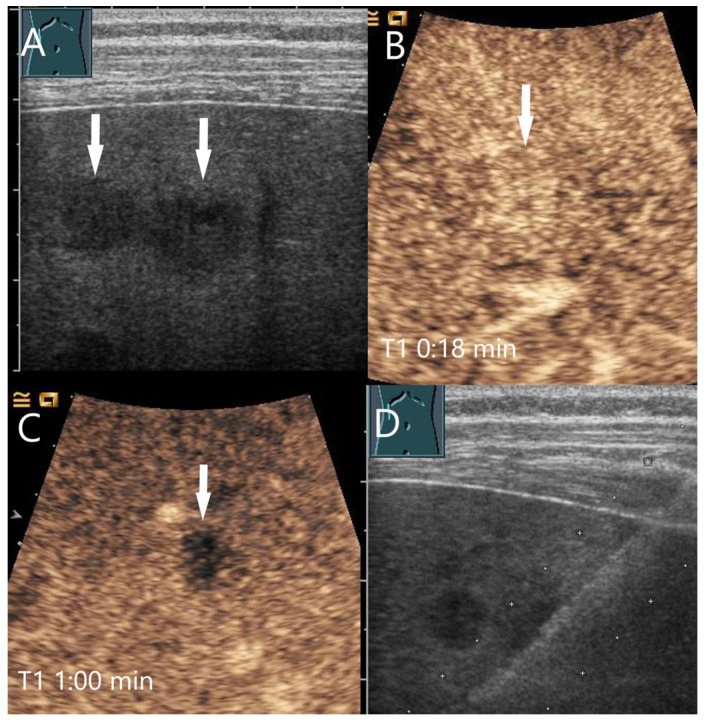
Mycotic abscesses. Acute myeloid leukemia, female patient, condition after neutropenia under antimycotic therapy, now after regeneration of the bone marrow. B-scan US shows multiple hypoechoic FLLs with a maximum size of 10 mm (arrows) (**A**). CEUS shows a slight arterial hyperenhancement (arrow) after 18 s (**B**) with clear washout and hypoenhancement after 1 min in the PVP (**C**). The US-guided biopsy (**D**) revealed a diagnosis of inflammation without evidence of fungi, clinically corresponding to hepatic candidiasis.

Mycotic liver abscesses are described less frequently. They usually occur in immunocompromised patients [77]. They occur predominantly in the regeneration phase after neutropenia. Histological detection of fungi is rarely successful, as antifungal therapy is carried out as standard in neutropenia. The final diagnosis is made by imaging with corresponding clinical findings [78]. Candida, Aspergillus, Cryptococcus neoformans, and Histoplasma capsulatum were the main frequent pathogens of fungal infections. The lungs, liver, and spleen are the most common organs affected by invasive multilocular fungal infection [79]. In a study of various liver abscesses, the mucoid abscesses were characterized by a hyperechoic inhomogeneous parenchyma [80]. CEUS showed small hyperenhanced lesions with perilesional parenchyma hyperenhancement during the AP and early washout of the small lesions during the venous phase. In addition to medical history, the hyperemia of the surrounding liver parenchyma may be indicative of inflammatory lesions [77,80]. Otherwise, the lesions would also be suspicious for metastases. Görg et al. defined three types of rim enhancement of mycotic abscesses. One of the images shows a type III with homogeneous hyperenhancement after 4 s, then at 12 s, a central hypoenhancement (washout) [78]. Mycotic abscesses are presented in Figure 10.

#### 4.4.3. Actinomycetes Abscesses

Actinomycosis is a rare form of chronic, granulomatous infection caused by Gram-positive anaerobic bacteria of the Actinomyces species. Hepatic manifestation is rare and usually secondary to abdominal infection. Actinomycetes also occur as apathogenic microbes in the oral cavity and gastrointestinal tract. Frequent manifestation of the disease is the ileocecal region or the presence of an intrauterine device (IUD). The infection leads to abscesses, fistula formation, fibrosis, and adhesions [81,82].

**Figure 11:**
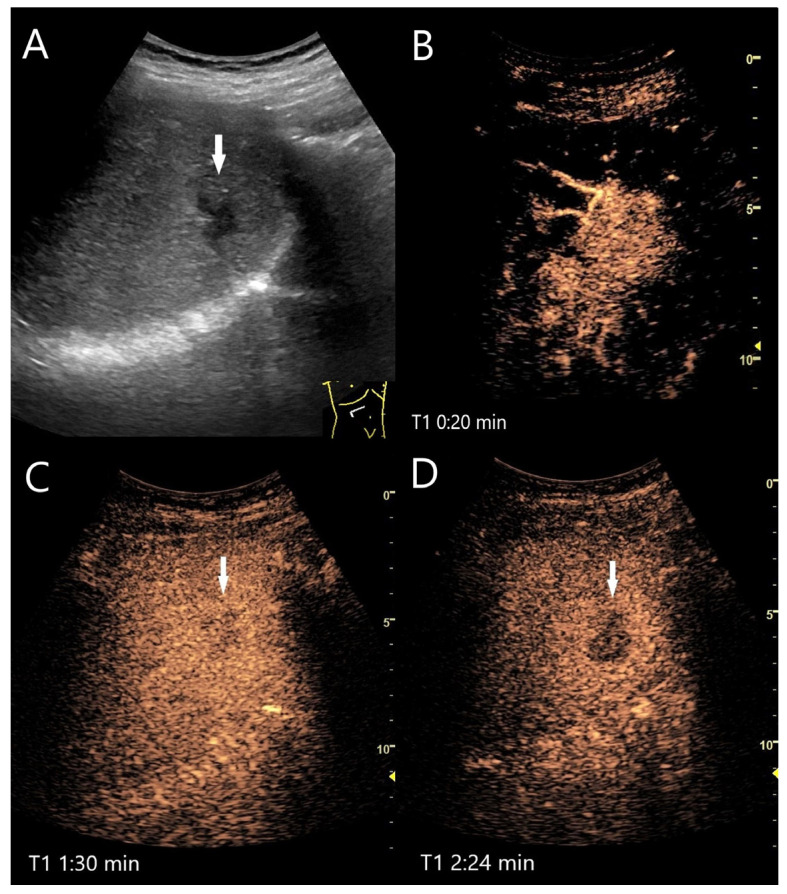
Actinomycetes abscesses. In a patient with chronic calcifying pancreatitis and weight loss, multiple hypoechoic lesions with a maximum size of 20 mm were found in the liver (arrow). These lesions were suspicious for metastases on both ultrasound and CT. Initially unnoticed, the surrounding area was slightly more echoic (**A**). On CEUS, the lesions were hyperenhanced, including the slightly hyperechoic surroundings in the AP (**B**). In the PVP, a washout of the central parts began, while the surrounding area remained slightly hyperenhanced (**C**). In the LP, the central parts were clearly hypoenhanced (**D**). US-guided biopsy was performed under suspicion of metastases. The diagnosis of actinomycetes abscesses was made in the biopsy specimens.

There are few case reports describing the appearance on CEUS where the lesions were hyperenhanced in the AP, followed by washout in the PVP. There was also a slight hyperenhancement of the surrounding liver parenchyma in the AP as an expression of inflammatory hyperemia [83]. This may indicate that it is not metastasis but an inflammatory process. The lesions could be misinterpreted as metastases or primary liver tumors or abscesses of more common bacterial spectrum (Figure 11). The diagnosis is based on the detection of the pathogen in the biopsy specimen. 

#### 4.4.4. Parasitic Abscesses

##### Toxocariasis (Visceral Larva Migrans)

Non-specific characteristics are hepatomegaly, lymphadenopathy, and pleuropericardial effusions. Chaubal et al. [77] described toxocariasis liver abscesses as multiple small hypoechoic lesions [77]. These have the typical abscess characteristics on CEUS: hyperenhancement in the AP with hypoenhancement in PVP and LP [84].

##### Fasciolosis Hepatica

Fasciola hepatica (and also clonorchiasis) appears in B-mode US as a hypoechoic FLL with blurred borders. There were also enlarged lymph nodes in the hepatic hilus. CEUS showed a hyperenhanced surrounding parenchyma around non-enhanced areas with washout and hypoenhancement in the LP [85,86]. The diagnoses were confirmed serologically.

##### Paragonimus

Paragonimiasis is a parasitic infestation caused by the lung fluke. Hepatic manifestation is also possible and manifests with hypoechoic lesions on B-mode US. On CEUS, the lesions show the characteristics of inflammatory lesions/abscesses with hyperemia, non-enhancement of necroses, enhanced capsules, and septa [87]. 

##### Amebic Abscesses

**Figure 12:**
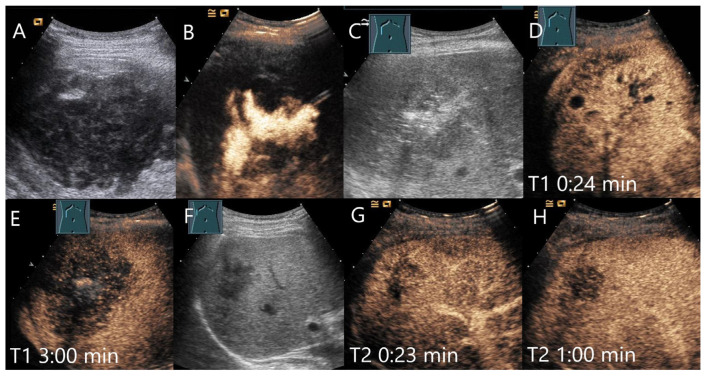
Female patient with amebic abscess, fever, and general malaise after a stay in northern India. On B-mode US, a 90 × 65 mm large liquid hypoechoic, well demarcated FLL (**A**). The abscess was relieved by drainage and the position was checked by intracavitary CEUS (**B**). After complete drainage of the liquid contents, a hypoechoic lesion remained (**C**). The liver tissue surrounding the heterogeneous enhanced lesion showed a large area of arterial isoenhancement after 24 s (**D**). The lesion is hypoenhanced after 3 min with hyperenhanced surrounded parenchyma (**E**). Serology revealed an elevated amebic titer. Under appropriate therapy, regression occurred after 4 weeks (**F**). However, the lesion showed a hypoenhancement after 23 s (**G**) and progressive washout after 1 min (**H**).

Amebic abscesses have a similar appearance in B-mode US [88]. We are only aware of one case report describing an amoebic abscess in the CEUS [89]. An amebic abscess on B-mode US and CEUS is shown in Figure 12.

#### 4.4.5. Granulomatous Inflammation

Granulomatous liver disease can have a variety of causes: manifestation of localized liver disease or a part of a systemic process, usually infectious or autoimmune. Causes of epithelioid necrotizing granuloma include tuberculosis, nocardiosis, and fungal infections. Epithelioid non-necrotizing granuloma includes sarcoidosis, hepatitis C, primary biliary cirrhosis (PBC), and drug induced liver injury (DILI) [90]. The diagnosis is usually made by liver biopsy and histology.

##### Sarcoidosis

**Figure 13:**
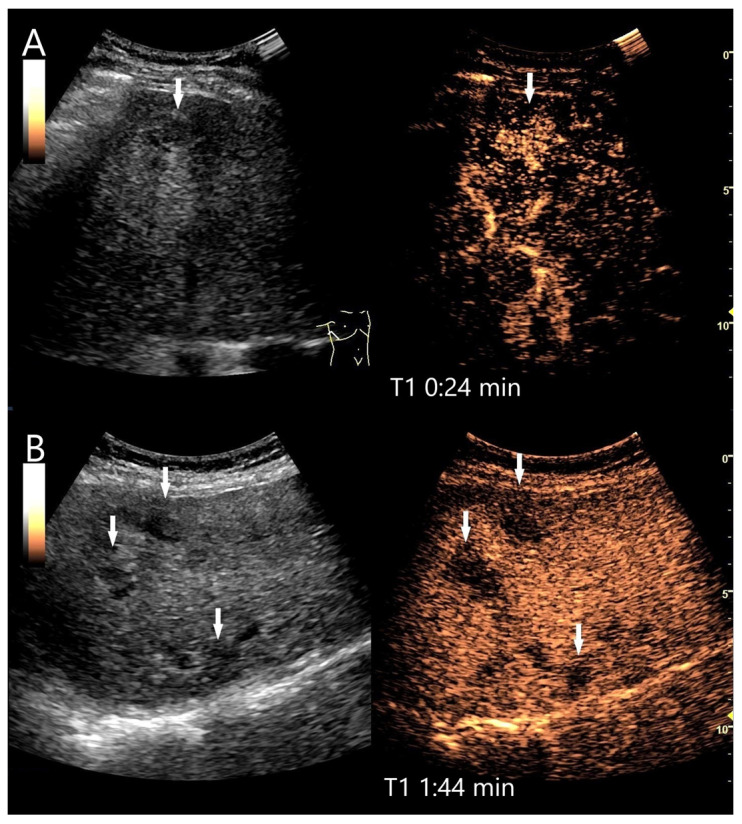
Hepatic sarcoidosis. Hepatosplenomegaly, up to 15 mm hypoechoic lesions in the liver and spleen, generalized lymphadenopathy, and poor general condition. On CEUS, these liver lesions are homogeneously hyperenhanced in the AP (arrow) (**A**). Washout with hypoenhancement develops from the PVP onwards (arrow) (**B**). Figure (**A**,**B**) show the CEUS image with the corresponding B-mode image in low MI mode. The diagnosis of the hepatic manifestation of sarcoidosis was made by US-guided biopsy of the liver.

**Figure 14:**
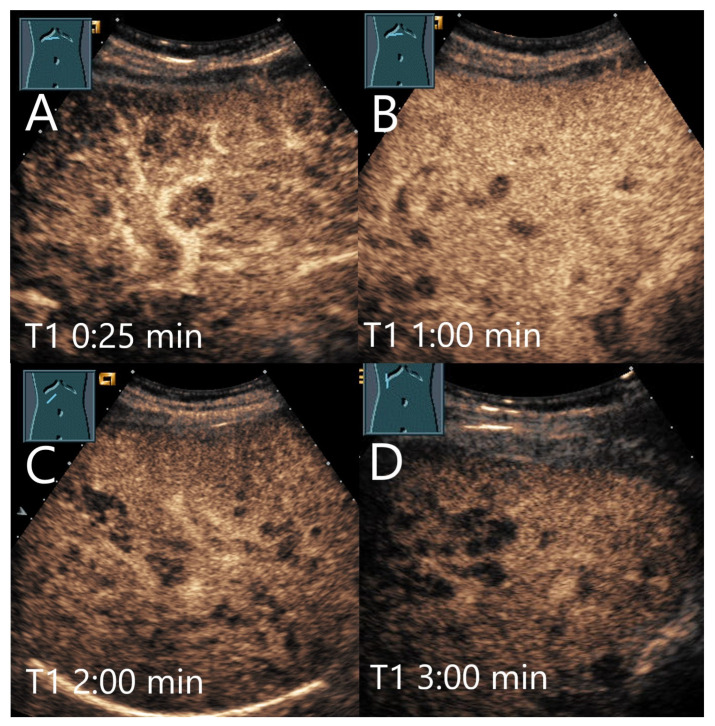
Sarcoidosis. Female patient with multiple hypoechoic liver lesions up to 10 mm. The CEUS shows after 25 s (**A**), 1 min (**B**), 2 min (**C**), and after 3 min (**D**) a progressive hypoenhancement. The US-guided biopsy revealed a diagnosis of hepatic sarcoidosis.

**Figure 15:**
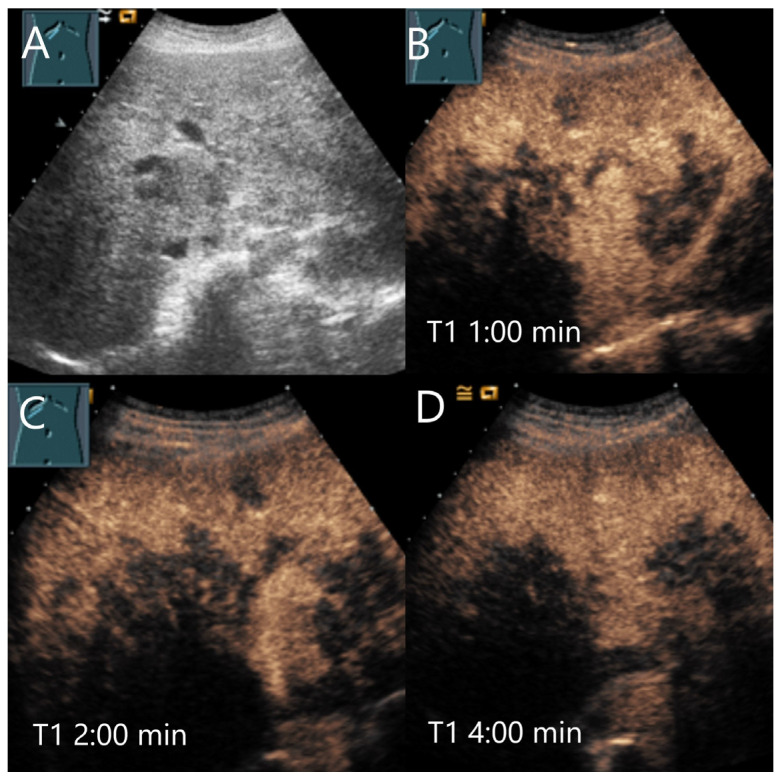
Necrotizing sarcoid granulomatosis (NSG) (special form of sarcoidosis characterized by granulomatous vasculitis of the pulmonary veins and pulmonary arteries). Female patient. Multiple confluent hypoechoic focal FLLs (**A**). CEUS shows progressive hypoenhancement after 1 min (**B**), 2 min (**C**), and after 4 min (**D**). The US-guided biopsy revealed a diagnosis of liver involvement in NSG.

Sarcoidosis is a complex granulomatous disease that can affect many organs, not just the lungs and lymph nodes [91]. Liver involvement is reported in up to 20% of patients. In micronodular changes, imaging shows no or only non-specific changes, such as hepatomegaly, hyperechoic parenchyma, and coarse nodular pattern. Rather rarely, focal hypoechoic lesions are detectable. There are only casuistic reports for sarcoidosis lesions in the liver. On CEUS, these hypoechoic lesions are characterized as differently arterially enhanced and progressive hypoenhanced lesions in the PVP and LP (Figure 13, Figure 14 and Figure 15). Persistent hyperenhancement is also described [91,92]. The progressive hypoenhancement can be a differential diagnostic aspect of malignancy. Completely non-enhancing (avascular) lesions in patients with long-standing sarcoidosis have also been described. Histologically, non-caseating granulomas were diagnosed [93]. Mediastinal lymphadenopathy, lung and spleen infiltrations can be diagnostically helpful. Splenic lesions in sarcoidosis behave similarly with progressive washout and hypoenhancement in the parenchymal phase of the spleen [94,95,96]. Histological confirmation by US or EUS-guided biopsy with detection of non-caseating granulomas is indicative. For differential diagnosis of granulomatous disease, see also the next subsection on tuberculosis.

##### Tuberculosis

Liver involvement with active tuberculosis is very rare (1%) but is reported much more frequently with simultaneous HIV injection (18%) [97,98,99,100]. Tuberculosis manifestations in the liver show a very different appearance in B-mode US, miliary, or macronodular lesions or the serohepatic form with thickened liver capsule and subcapsular lesions [101,102,103,104,105,106,107]. Depending on the stage of the disease, the lesions in the CEUS may be homogeneously hyperenhanced in the AP and most lesions develop a washout in the PVP. Caseous abscesses have a hyperenhanced rim, with either hypo- or isoenhancement in the center. The morphological correlation is granulomatous inflammation with central caseous necrosis and peripheral granulation tissue [83,101,108]. 

In a study with 22 lesions [101], 54% showed a hyperenhanced rim with a hypoenhanced or non-enhanced center in the AP in CEUS, while in 37%, the lesion was transiently completely hyperenhanced. In most lesions, washout occurred in the PVP. The enhancement of the hyperenhanced rime decreased in the PVP in the majority of cases and was hypoenhanced in 46% of cases. Histopathological examination by needle biopsy revealed that the hyperenhanced rim in the CEUS was due to inflammatory hyperemia. An increased number of leukocytes and lymphocytes were detected in the hepatic sinusoids. As a correlation to the hypo- and non-enhanced central areas, epithelioid granulomas with incomplete or complete caseous necrosis or liquefaction were found. A washout of the contrast medium was observed in the central parts of the lesions during the PVP. The histological correlate of this was again destruction of the hepatic sinusoids with inflammatory granulation, fibroplasia, epithelioid granuloma, Langerhans giant cells, and lymphocyte infiltration [101]. Tuberculosis manifestations in the liver may resemble liver metastases as well as primary liver malignancies. There are no characteristic features. However, in endemic areas, medical history and the overall constellation may give rise to suspicion. Diagnosis can be made by US or CEUS-guided needle biopsy to obtain material for histological evaluation, culture, and tuberculosis polymerase chain reaction (PCR). The diagnosis should be specific to tuberculosis [109,110].

#### 4.4.6. Inflammatory Pseudotumor

Inflammatory pseudotumors (IPTs) are tumor-like lesions associated with both acute and chronic inflammation. IPTs can be classified into two types based on their clinicopathologic features: lymphoplasmacytic and fibrohistiocytic types [111]. The lymphoplasmacytic type could belong to the so-called IgG4-related diseases. In contrast, the fibrohistiocytic type could still represent a heterogeneous group of diseases [111]. IPTs in B-mode are usually hypoechoic, homogeneous, or inhomogeneous. In CEUS, they are arterially hyperenhanced with hypoenhancement in the LP, often with enhancement degree in the AP, early washout, peritumoral vessels, and peritumoral enhancement [112]. Isoenhancement in the AP with subsequent washout makes differentiation from malignant tumors difficult and requires histological confirmation [113,114]. IPTs can also occur in multiple locations in the liver and mimic liver metastases with [115]. Kong et al. [116] described 44 patients with inflammatory pseudotumors. In the AP, there were three types of enhancement patterns: homogeneous, heterogeneous, and rim enhancement. All IPTs showed hypoenhancement in the PVP and LP. Rapid washout began in 29% before 60 s [116]. The smaller the IPT, the earlier the washout began in one study [117]. In a study from two centers with 83 hepatic IPTs, 61.5% showed mild hyper- or isoenhancement in the AP, 59% internal non-enhanced areas, and 71% an early washout <60 s [118]. A centrally emphasized enhancement in the AP in CEUS are described, followed by washout in the PVP [119].

**Figure 16:**
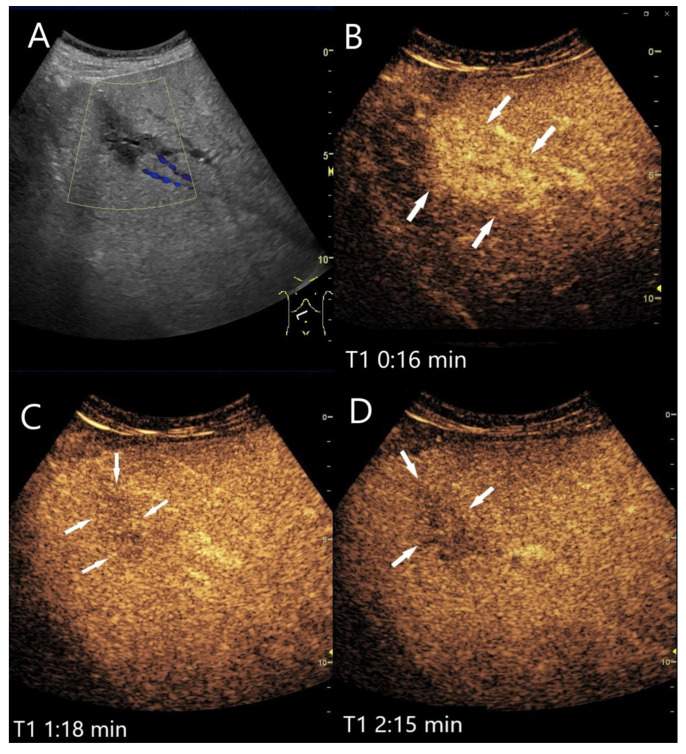
IgG4-associated inflammatory pseudotumor. In a patient with elevated liver enzymes and alcohol abuse, B-mode US showed steatosis hepatis and a 55 × 20 mm irregular oval hypoechoic lesion (**A**). The central tubular structure was without flow evidence on CDI, and we interpreted this as a small bile duct branch. On CEUS, the lesion (arrows) was homogeneously hyperenhanced in the AP (**B**). In the PVP, a mild hypoenhancement developed (**C**), which became more pronounced in the LP (**D**).

**Figure 17:**
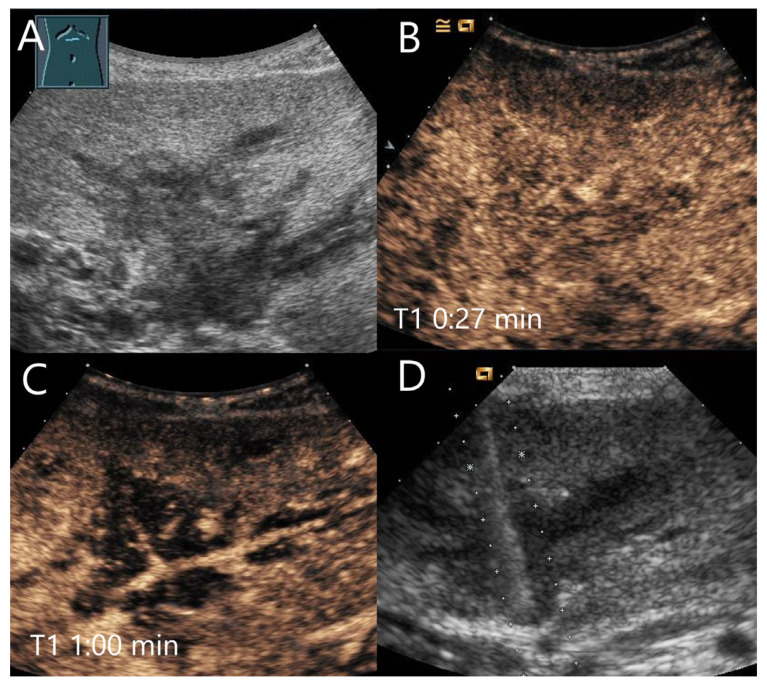
IgG4-associated inflammatory pseudotumor. Male patient with pain in the right upper abdomen. The B-mode US shows a hypoechoic wall around the portal veins in the center of the hepatic hilus (**A**). CEUS shows a slight arterial hypoenhancement after 27 s (**B**) with clear parenchymal washout after 1 min in the PVP (**C**). This irregularly delimited area had a size of about 60 × 50 mm. The US-guided biopsy (**D**) was diagnostic for IgG4 positive chronic sclerosing lymphoplasmacytic inflammation.

The enhancement of an IPT depends on the composition of the tumors. Arterial hyperenhancement can be explained by inflammation. Varying degrees of fibrosis and cellular infiltration may account for the different various enhancement manifestations [117]. As with other inflammatory lesions, it can be assumed that obliterative phlebitis is due to an inflammatory infiltration of the vessel walls and lumina and thrombosis are the cause. In the fibrohistiocytic type, venous occlusion with little inflammation and cholangitis without periductal fibrosis were frequently observed, whereas obliterative phlebitis and cholangitis with periductal fibrosis were described in the lymphoplasmacytic type [111]. Depending on the appearance in the AP, other important differential diagnoses are HCC, liver metastases, and cholangiocellular carcinomas (Figure 16 and Figure 17).

### 4.5. Angiomyolipoma, Perivascular Epithelioid Cell Neoplasms (PEComas), and Epithelioid Angiomyolipomas (EAML)

Hepatic angiomyolipomas, perivascular epithelioid cell neoplasms (PEComas), and epithelioid angiomyolipomas (EAML) are mesenchymal tumors [120,121,122,123,124]. The EAML is a variant of the classic angiomyolipoma, which differs in its distinct epithelioid cell components. Some authors describe that perivascular epithelioid cell neoplasms (PEComas) and epithelioid angiomyolipomas (EAMLs) are two different terms for the same “mesenchymal tumor composed of histologically and immunohistochemically characteristic perivascular epithelioid cells” [125]. In the WHO classification of mesenchymal tumors, all three entities are classified as tumors of uncertain differentiation [126]. However, PEComa and AML are classified as benign tumors and EAML as intermediate (locally aggressive) tumors [126]. The distinction between PEComa and EAML in the literature and case reports sometimes appears somewhat indistinct.

#### 4.5.1. Hepatic Angiomyolipoma (HAML) 

HAML is a rare mesenchymal liver tumor comprising smooth muscle cells, adipose tissue, and thick-walled blood vessels. HAML has no typical clinical or sonographic appearance but is well vascularized. This makes it necessary to differentiate it from other well-vascularized FLLs and HCC. In a study of Zhang et al. [120], 38 HAMLs showed a variable appearance in B-mode: most frequently hyper- and hypoechoic separation and hyper- or hypoechoic lesions. Specific was only the relatively strong hyperechogenicity with attenuation. Using CEUS with SonoVue, 50% of the HAMLs showed a washout that never started before 60 s. In 42.1% of all HAMLs, the washout onset started after 120 s. The observation period was up to 5 min during the examination with SonoVue. The washout was mostly (36.8% of all HAMLs) a mild washout; in 5.3%, a marked washout was reported and in 7.9% a partial washout with partial no washout was reported [120]. In a study with 33 HEAMLs, 9/33 (28%) showed hypoenhancement, which was only detectable from 120 s onwards [127]. These data differ from a previous study [128] in which 16/17 arterially hyperenhanced HAMLs remained hyperenhanced in the LP and only one HAML developed isoenhancement. Only two lesions were hypoenhanced in the AP, PVP, and LP. [128]. The technical data of the studies did not differ significantly. The examination period lasted up to 5 min, an MI of 0.19 [120], <0.14 [127], or <0.2 [128]. Selected SonoVue dose was 1.5–2.0 mL [120], 1.5–2.4 mL [127], and 2.4 mL [128], respectively. 

#### 4.5.2. Perivascular Epithelioid Cell Neoplasms (PEComas) and Epithelioid Angiomyolipomas (EAMLs)

**Figure 18:**
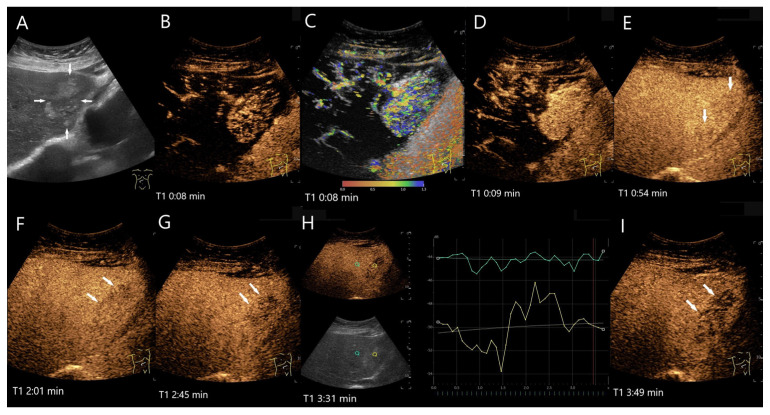
Hepatic PEComa. In the left lobe of the liver there is a more than 5 cm large, oval, irregularly demarcated heterogeneous lesion (arrows) with several hyperechoic areas (**A**). In the AP of the CEUS (**B**) and in parametric imaging (**C**), the enhancement is initially lateral. The parametric imaging shows the chronological sequence of the arrival of the UCA in color. Then, the lesion is homogeneously hyperenhanced (**D**). In the PVP before 60 s, the hypoechoic parts show mild hypoenhancement (arrows) (**E**). The hyperechoic parts are isoenhanced or even slightly hyperenhanced. The hypoenhancement is slightly more pronounced at the beginning of the LP (**F**). This increases in the course of the LP (**G**) and is objectified in the time intensity curve (**H**). In the advanced LP the lesion shows a significant hypoenhancement (**I**). The washout with hypoenhancement was the reason for the US-guided biopsy, which resulted in a PEComa. This was confirmed by the histology of the surgical specimen.

EAMLs are characterized by distinct epithelioid cell components. Adipocytes may also be found in varying proportions. Boccatonda et al. researched 29 case reports of hepatic PEComas and 25 of hepatic EAMLs [125]. Hepatic PEComas and EAMLs have a highly variable histological composition [122], and in case reports, the appearance of these tumors in B-mode US, CDI, and CEUS proved to be very variable [122,125,129]. The appearance on imaging depends on the proportion of different components (adipocytes, perivascular cells, and enlarged blood vessels) in the tumor [130]. EAMLs are typically described as homogenously hyperenhanced in the AP in the CEUS with a tendency towards observed centripetal enhancement [131,132]. Hypoenhancement in the LP has been described in 67% (4/6 patients) [131] and 37% (9/24 patients). A rapid washout occurred in 25% (6/24 patients) [132]. PEComas appear to be variable in the LP, usually without marked washout [129]. Central washout of PEComas in the LP has been described in single cases [129,133]. Dong et al. [134] illustrated a PEComa that shows a homogeneous, pronounced hyperenhancement after 20 s and is still slightly hyperenhanced after 4 min. Only after 6 min does the lesion demonstrate a slight hypoenhancement [134]. This was only recorded due to the long observation period. The EAML contains differently dilated and distorted vascular networks, which causes hyperenhancement in the AP [122,132]. Huang et al. suggested a direct outflow of arterial blood into the hepatic vein branch “causing a short circuit in the hepatic artery-portal vein” [132]. In another study, 12/12 (100%) of the EAMLs showed hyperenhancement in the AP and in 10/12 (83%) hypoenhancement in the LP, which developed in 4/12 (33%) in the PVP. The authors found it difficult to differentiate between EAML and AFP-negative HCC [135]. EAML and PEComa are mostly benign, but rare malignant tumors have been described [136,137]. In the classification according to Folpe et al. [138], PEComas with a size of >5 cm, vascular infiltration, a proliferation index of >1/50 HPF (high power fields), and tumor necrosis have a higher risk of malignancy and should be surgically resected [138]. Important differential diagnoses of other well-vascularized FLLs with washout are HCCs, well-vascularized metastases, and HCAs. The diagnosis is usually made histologically by biopsy or surgical resection (Figure 18).

### 4.6. Lipoma

Liver lipomas are rare. In an ultrasound series of five solitary liver lipomas, these were homogeneous in B-mode US, hyperechoic and with smooth margins [139]. In a CEUS study of FLLs [140] including a single hyperechoic hepatic lipoma, this showed homogeneous hyperenhancement after 34 s. The slight washout started in the PVP, and hypoenhancement was described after 180 s. In the DEGUM multicenter study [16] with workup of 86 FLLs that were not diagnostic in the CEUS, a lipoma with LP hypoenhancement is listed.

### 4.7. Peliosis

Peliosis hepatis (PH) is a benign condition that is histologically characterized by a proliferation of sinusoidal hepatic capillaries with blood-filled cystic cavities of varying size and irregular shape. Hepatic peliosis is usually asymptomatic. However, spontaneous bleeding may occur [141,142]. Post sinusoidal obstruction is suspected as a possible mechanism [143,144]. An altered venous outflow tract is discussed as the cause. However, the cause is often unclear. Chronic wasting diseases are frequently associated with peliosis, as are various *Bartonella* species. *Bartonella henselae* and *Bartonella quintana* cause bacillary peliosis in patients with AIDS [145,146]. In 2632 patients with newly diagnosed liver lesions and history of colorectal carcinoma, 9 (0.3%) had peliosis hepatis [147]. 

Hepatic peliosis is often an arterial hyperenhanced lesion that shows a washout in the PVP or LP on CEUS. A biopsy of these lesions is associated with a high risk of bleeding [148]. In a study of 24 patients, the hepatic peliosis lesions were mostly solitary, but also multiple. In B-mode US, the lesions appeared heterogeneously hypoechoic with well-defined margins but irregular shapes. On CEUS, 83% of the lesions showed a slight heterogeneous hyperenhancement in the AP, 12.5% a centrifugal hyperenhancement, and 16.7% an isoenhancement. Moreover, 87.5% of the lesions developed a slight washout with hypoenhancement in the PVP after 60 s and LP [141]. Further case reports reported hypoenhancement [149] or central hypoenhancement in the LP [150,151].

In another case report, an arterial homogeneous hyperenhancement was described, followed by an early progressive washout from 40 s [143]. The authors compared this appearance on CEUS with that of liver metastases or cholangiocellular carcinoma [143]. However, the washout does not appear to be obligatory. In another case with histologic confirmation of peliosis, the lesion showed central enhancement in the early AP with centrifugal spread and homogeneous enhancement in the LP [152].

In the study by Dong et al. [141], the typical histopathological features of parenchymal peliosis in the majority of patients were the presence of localized irregular sinusoids forming blood-filled spaces in the liver parenchyma, with thinning of the hepatic cell strands and the reticulin fiber network. In patients with the phlebectatic variant of peliosis, the presence of an endothelial lining along the blood-filled spaces caused by aneurysmal dilatation of the central vein was observed. In 28% of patients, the lining was secured by liver surgery. In all others, a needle biopsy was performed, with no major complications [141].

**Figure 19:**
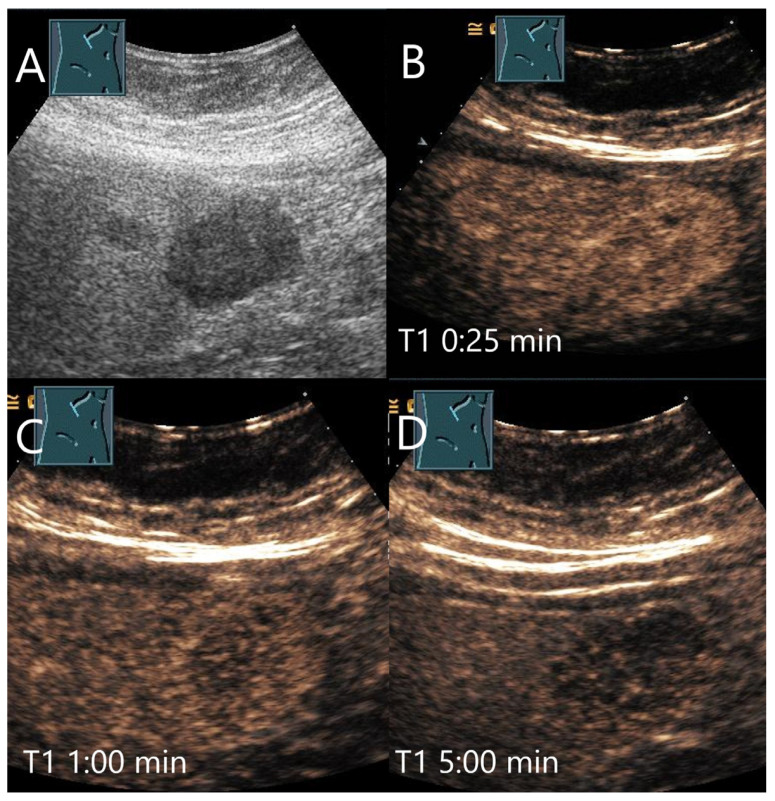
Peliosis hepatis. Female patient. Hypoechoic lesion of 40 × 28 mm in a mildly steatotic liver (**A**). The CEUS shows arterial hyperenhancement after 25 s (**B**) with mild predominantly central hypoenhancement after 1 min (**C**) and 5 min (**D**). Histological confirmation was performed by US-guided biopsy. The adjacent small hypoechoic lesion (**A**) is isoenhanced in the CEUS in all phases.

Ultimately, the final diagnosis of hepatic peliosis can usually only be confirmed histopathologically. Typical features are dilated sinusoidal spaces and hemorrhagic dilated spaces within the liver parenchyma, but this requires adequate tissue sampling. This may not be feasible in dilated sinusoidal spaces. In addition, a high risk of bleeding should always be taken into consideration with hepatic peliosis. However, the aspirates could also be examined for *Bartonella* species (Figure 19).

### 4.8. Cholangiocellular Adenoma

**Figure 20:**
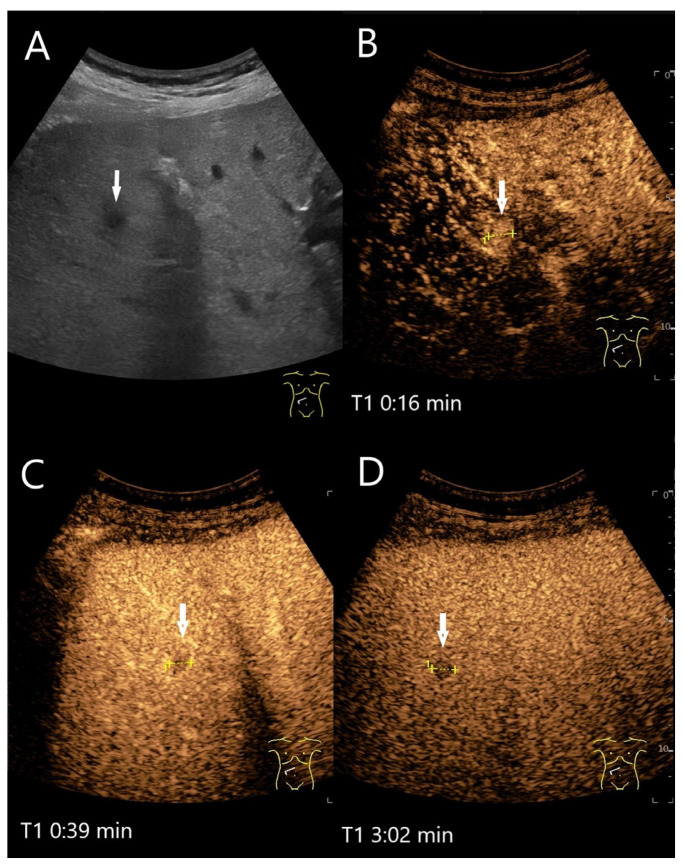
Cholangiocellular adenoma. Male patient with elevated transaminases. A small, hypoechoic lesion < 10 mm (arrow) is seen in B-mode US (**A**). In the AP of CEUS, the lesion (between the markings) is homogeneously hyperenhanced (**B**). In the PVP < 60 s (**C**) and in the LP (**D**), the lesion is hypoenhanced. US-guided biopsy was performed under suspicion of a malignant lesion. However, the histology revealed a cholangiocellular adenoma. Surgical resection was performed with confirmation of a benign cholangiocellular adenoma.

**Figure 21:**
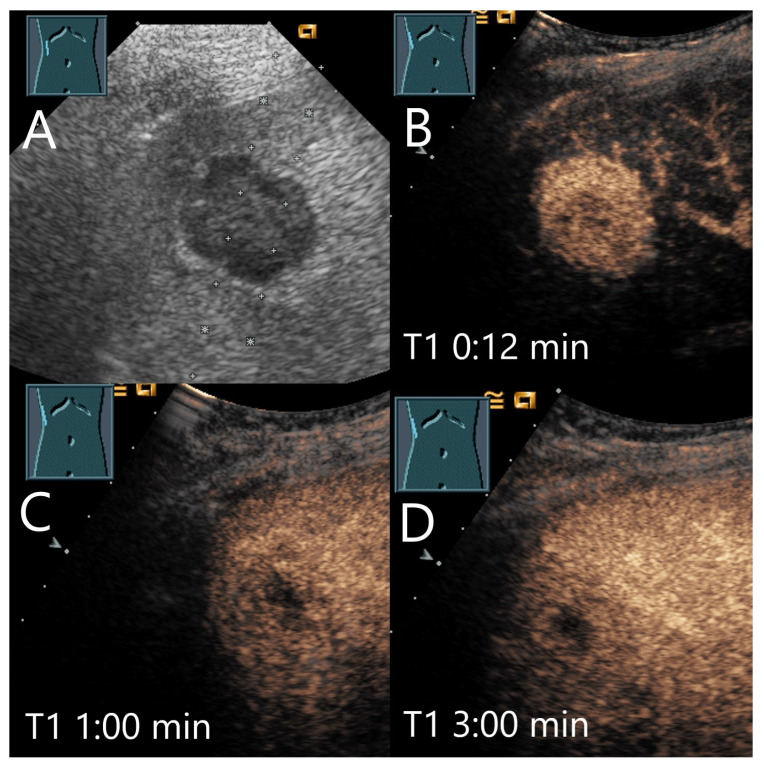
Sclerosing bile duct adenoma. Female patient with a 31 × 27 mm hypoechoic round liver lesion as an incidental finding (**A**). The CEUS shows arterial hyperenhancement after 12 s (**B**) with hyperenhancement with mild predominantly central hypoenhancement after 1 min (**C**) and 3 min (**D**). Histological confirmation was performed by US-guided biopsy with histologic diagnosis of a benign sclerosed bile duct adenoma without relevant somatic mutations. This was confirmed by histology after surgical resection.

Cholangiocellular adenoma is a rare tumor. The histological correlation is the disorganized proliferation of small, non-cystic bile ducts lined by cuboidal cells without nuclear atypia and associated with varying degrees of fibrosis and inflammation. These are mostly single, small lesions < 10 (up to 20) mm and subcapsular located, often incidental findings intraoperatively or at autopsy [153]. Ignee et al. described three lesions, well circumscribed and hypoechoic (two in non-steatotic liver). We only found the CEUS description of Ignee et al. In the AP, both hyper- and isoenhancement were observed with marked washout in the LP [154]. Bile duct adenoma in the common bile duct is similarly described in CEUS with APHE and washout during PVP and LP with LP hypoenhancement [155,156]. The contrast behavior can apply to both benign and malignant biliary tract obstructions. We demonstrate cholangiocellular adenomas in Figure 20 and Figure 21.

### 4.9. Extramedullary Hematopoiesis

**Figure 22:**
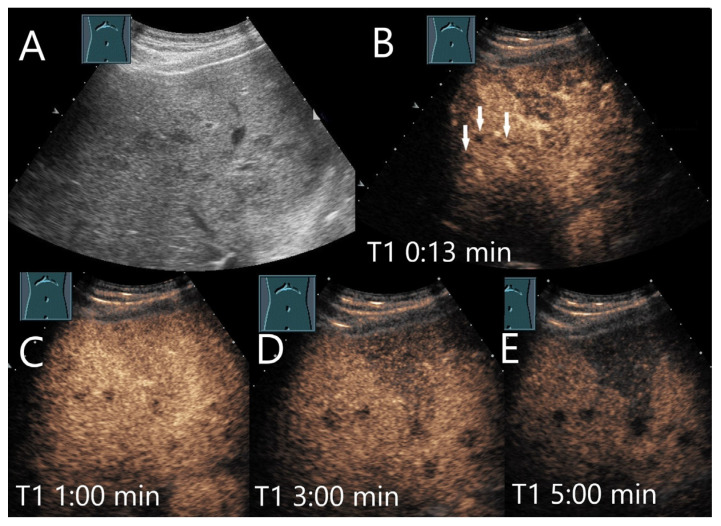
Extramedullary hematopoiesis. Male patient with myelodysplastic syndrome and secondary acute myeloid leukemia and multiple hypoechoic lesions (arrows) (**A**). CEUS shows inhomogeneous arterial liver enhancement after 13 s (**B**) with slight hypoenhancement after 1 min (**C**) and increasing hypoenhancement after 3 min (**D**) and 5 min (**E**). In addition, a wedge-shaped lesion with slight hypoenhancement is demarcated. US-guided biopsy revealed the diagnosis of extramedullary hematopoiesis.

Usually extramedullary hematopoiesis (EMH) is a compensatory mechanism as a result of bone marrow dysfunction. The most common cause is myelofibrosis [157]. Extramedullary hematopoiesis foci mostly form in the liver and spleen, but other organ manifestations are also possible. EMH can manifest itself with hepatosplenomegaly [158]. Hypoechoic, hyperechoic, and isoechoic lesions have been described [159]. Alhyari described an extramedullary hematopoiesis, which was not distinguishable in the B-mode, and hypoenhanced lesions only appeared in the LP [160]. EMH lesions are tissue foreign to the liver (Figure 22).

## 5. Summary of the Typical Appearance of Benign FLL and Possible Causes of Washout 

The assessment of FLL should always consider all anamnestic data, physical complaints, and laboratory parameters. B-mode US detects the lesions and provides important information. However, further clarification is carried out using CEUS and/or radiological cross-sectional imaging (contrast-enhanced magnetic resonance imaging and computed tomography). The particular enhancement pattern provides important information that often enables the diagnosis of the lesion type on CEUS. The differential diagnosis of well-vascularized FLLs in the non-cirrhotic liver can be difficult. A frequent question in clinical routine at CEUS is the differential diagnosis between FNH and HCA, with additional exclusion of HCC. We find it particularly difficult to differentiate between the telangiectatic variant of FNH and I-HCA. In case of doubt, a US-guided biopsy must be performed. Table 5 summarizes important characteristics of FLLs in B-mode US, CDI, and CEUS and the possible causes of LP hypoenhancement. The CEUS appearance of many rare liver lesions is illustrated in a series of the WFUMB “Comments and illustrations of the WFUMB CEUS liver guidelines”. See https://wfumb.org/publications/focal-liver-lesions/.

**Figure 23:**
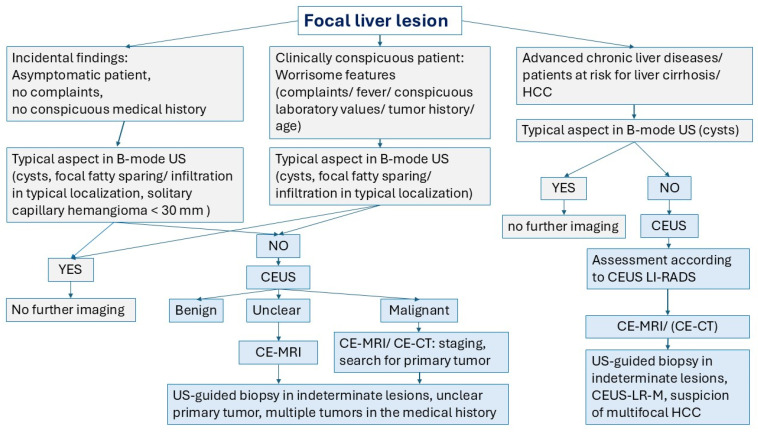
Algorithm for the clarification of FLL.

In Figure 23, we propose an algorithm for the clarification of FLL depending on the clinical situation of the patients.

## 6. Conclusions

Washout and hypoenhancement in the LP of CEUS are the hallmarks for the diagnosis of malignant FLLs but can potentially also occur in all benign FLLs entities for various reasons. The washout with the development of hypoenhancement can occur in the different vascular phases, at different times, and with varying intensity on CEUS. Washout is an atypical feature in FNHs and hemangiomas. In FNHs, it results from the regression of the FNH with vascular obliteration and usually simultaneously shrinking over the course of many years of follow-up. If a FNH shows a typical appearance at the time of diagnosis and shows regressive changes and washout on repeat imaging over the years, this is no cause for concern. If the initial diagnosis shows a wheel-spoke-like centrifugal enhancement, but then a washout with hypoenhancement, further clarification with MRI and US-guided biopsy should be performed. If hemangiomas are exposed to ultrasound too long and/or with an inadequate high mechanical index, the bubble destruction cannot be compensated by the slow inflow of fresh UCA microbubbles. For this reason, an intermittent examination should be carried out if a hemangioma is suspected. Slow flow with slow filling of the hemangioma, fibrosis, or drainage in connection with vascular fistulas are additional factors promoting washout in hemangiomas. One possibility is to perform a second application of contrast medium and to examine the suspected hemangioma exclusively and intermittently in the LP. If the enhancement in the AP is typically ring-shaped and globular, this can be regarded as evidence of a hemangioma. If the appearance in the AP is not typical and there is an APHE with washout in the LP, further diagnosis and US-guided biopsy should be performed. Due to a lack of portal veins, the majority of hepatocellular adenomas show a late slight washout. In inflammatory lesions, early washout is a typical and expected feature. This is due to pylephlebitis and vascular thrombosis in the lesions. Since HCAs show an APHE but no typical vascular architecture and the diagnosis is only presumptive, histological confirmation is required. 

For various abscesses, it is clinically appropriate to perform a drainage, and examination of the contents for pathogens and antibiotic resistance, but not a biopsy of the marginal parenchymal parts with hypoenhancement. An exception is the differential diagnosis of necrotic melting superinfected metastases, in which a primary tumor is usually known. 

If liver lesions occur with known sarcoidosis, it is obvious that this is a further manifestation. However, focal sarcoidosis liver lesions are rare and other differential diagnoses should be excluded by US-guided biopsy.

The diagnosis of FLLs is made in conjunction with the patient’s clinical situation and medical history. This is particularly important. Since washout and hypoenhancement in the LP of CEUS are typical criteria of malignant FLLs, this feature should be taken very seriously. It may be useful to perform magnetic resonance imaging (MRI), but an US-guided biopsy is always indicated if the findings are unclear and cannot be assigned to obtain a histological diagnosis. In particular, lesions without recognizable vascular architecture in the AP phase and with subsequent washout can only be clarified by biopsy and histology.

## Figures and Tables

**Table 1:** Phases in CEUS [1].

Phase	Start	End
Arterial (AP)	10–20 s	25–45 s
Portal venous (PVP)	20–45 s	≤120 s
Late (LP)	>120 s	Up to 4–8 min, depending on the presence of the UCA bubbles. With continuous sonication, the bubbles are destroyed prematurely.

**Table 4:** Inflammatory FLLs and hypoenhancement in LP.

Study	FLL (*n*)	LP Washout	Comments
Ding 2005 [52]	Benign lesions *n =* 51	*n =* 11/51 (22%)	
	Liver abscess *n =* 5	*n =* 3/5 (60%)	
	IPT *n =* 3	*n =* 3/3 (100%)	
Liu 2008 [65]	Inflammatory lesions *n =* 53		
	Pyogenic abscesses *n =* 32 (*n =* 31 with hyper- or isoenhancement in the AP)	*n =* 25/31 (80.6%)	In 54.8%, hypoenhancement started in PVP.
	Infected granulomas *n =* 15	*n =* 15/15 (100%)	In 100%, hypoenhancement started in PVP.
	IPT *n =* 6	*n =* 6/6 (100%)	In 100%, hypoenhancement started in PVP.
Bhayana 2010 [4]	Hypervascular benign FLL *n =* 74 (overall *n =* 146 FLL)	36% of all benign FLL	Washout occurred in 36% of benign and 97% of malignant FLL. The onset of washout after injection was defined as <30 s/after 30–75 s/75–180 s/<180 s.
	Inflammatory lesions *n =* 5	*n =* 5/5 (100%)	Mostly (80%) < 30 s.
Popescu 2025 [67]	Liver abscesses *n =* 41	*n =* 22/41 (53.6%)	Hypoenhancement in LP according to the marginal rim.
Guo 2020 [66]	Inflammatory lesions *n =* 44	*n =* 37/44 (84%)	Start of hypoenhancement in PVP *n =* 30/44 (68%).
Francica 2020 [68]	Liver abscesses *n =* 44	*n =* 30/38 (79%)	Data refer to peripheral hyperenhancing rim in AP.
		*n =* 10/20 (50%)	The data refer to hyperenhanced septa.

**Table 5:** Summary of typical characteristics of benign FLLs in B-mode US, CDI, and CEUS and causes of washout.

Lesion	Characteristics on B-Mode and CDI	Typical Characteristics on CEUS	PVP and LP	Explanation of Washout in LP
**FNH**	Isoechoic, hypoechoic, sometimes hypoechoic rim.	Wheel spoke pattern, central artery, rarely peripheral artery and wheel. spoke pattern. Centrifugal filling	Hyperenhancement to isoenhanced, central scare.	Fibrosis and vascular obliteration.
**Hemangioma**	Hyperechoic, beyond liver veins, hypoechoic in steatosis and with shunts.	Peripheral globular enhancement, centripetal filling. Rapid homogeneous filling in shunt hemangiomas.	Hyperenhancement and isoenhancement.	Permanent video loops with destruction of the UCA bubbles and slow refill. Fibrosis.
**HCA**	Hypo-/iso-/hyperechoic HNF1a HCA (steatotic HCA) are frequently hyperechoic.	centripetal or mixed/diffuse filling.	Iso- or late slight hypoenhancement. Hyperenhancement in some I-HCA.	Absence of portal and central veins.
**Abscesses**	Hypo-/anechoic.	Hyperenhancement in phlegmonous stage, transient hyperenhancement in surrounding parenchyma. Non-enhancement of necrotic parts. Honeycomb sign.	Early washout.	Formation of thromboses of the small hepatic and portal veins, as well as pylephlebitis of the small portalvenous vessels.
**Tuberculosis**	Miliary or macronodular lesions or the serohepatic form with thickened liver capsule and subcapsular lesions.	Hyperenhancement in granulomatous inflammation, hyperenhanced rim, and central hypoenhancement in caseous necrosis. Enhanced septa.	Hypoenhancement in the PVV.	Destruction of the hepatic sinusoids with inflammatory granulation.
**IPT**	Mostly hypoechoic, irregular shapes.	Arterial hyperenhancement, Homogenous, heterogenous, rim like.	Early washout.	Obliterative phlebitis is due to an inflammatory infiltration of the vessel walls and lumina and thrombosis, varying degrees of fibrosis.
**Cholangiocellular adenoma**	Small hypoechoic lesions, well circumscript.	Hyper- or isoenhancement.	Marked washout in the LP.	No liver tissue.
**Peliosis**	Heterogeneously hypoechoic, well-defined margins, irregular shapes	Often hyperenhanced in the AP	Hypoenhancement in PVP or LP	Post sinusoidal outflow obstruction.
**HAML**	Variable appearance, hyper- and hypoechoic separation specific is the strong hyperechoic appearance with attenuation.	Homogeneous or inhomogeneous hyperenhancement in AP, no or mild washout, hyperenhancement is described. Partial washout and non-washout in hyperechoic-hypoechoic separation.	Hypoenhancement not before 60 s or late after 120 s, mostly slight hypoenhancement, isoenhancement, and hyperenhancement is described.	
**PEComa/** **HEAML**	Variable appearance, peripheral vessels in CDI in PEComas.	Arterial hyperenhancement. Variable appearance in PVP and LP.	No marked washout.	Dilated and distorted vascular networks, a direct outflow of arterial blood into the hepatic vein branch “causing a short circuit in the hepatic artery-portal vein” is suggested.
**Extramedullary hematopoiesis**	Hepatosplenomegaly, hypoechoic, hyperechoic, isoechoic lesions.	No information.	In our case, hypoenhancement in the LP.	Non-hepatic tissue.

## List of Abbreviations

AP	arterial phase
APHE	arterial phase hyperenhancement
ß-HCA	ß-catenin mutated hepatocellular adenoma
CDI	color Doppler imaging
CE-CT	contrast-enhanced computed tomography
CE-MRI	contrast-enhanced magnetic resonance imaging
CEUS	contrast-enhanced ultrasound
DEGUM	German society of ultrasound in medicine
DILI	drug induced liver injury
EAML	epithelioid angiomyolipoma
EMH	extramedullary hematopoiesis
EUS	endoscopic ultrasound
FLL	focal liver lesion
FNH	focal nodular hyperplasia
HAML	hepatic angiomyolipoma
HCA	hepatocellular adenoma
H-HCA	hepatocyte nuclear factor 1-α HCA
HCC	hepatocellular carcinoma
IgG4	immunoglobulin G4
I-HCA	inflammatory HCA
IPT	inflammatory pseudotumor
LP	late phase
MI	mechanical index
NRH	nodular regenerative hyperplasia
NSG	necrotizing sarcoid granulomatosis
PBC	primary biliary cirrhosis
PEComa	perivascular epithelioid cell neoplasm
PH	peliosis hepatis
p.i.	post injection
PVP	portal venous phase
sh-HCA	sonic hedgehog activated HCA
T1	timer 1
T2	timer 2
UCA	ultrasound contrast agent
U-HCA	unclassified HCA
US	ultrasound
